# Lateral Mesoderm-Derived Mesenchymal Stem Cells With Robust Osteochondrogenic Potential and Hematopoiesis-Supporting Ability

**DOI:** 10.3389/fmolb.2022.767536

**Published:** 2022-04-28

**Authors:** Yili Wei, Bin Wang, Lei Jia, Weijun Huang, Andy Peng Xiang, Cong Fang, Xiaoyan Liang, Weiqiang Li

**Affiliations:** ^1^ Center for Stem Cell Biology and Tissue Engineering, Key Laboratory for Stem Cells and Tissue Engineering, Ministry of Education, Zhongshan School of Medicine, Sun Yat-Sen University, Guangzhou, China; ^2^ Reproductive Medicine Research Center, Sixth Affiliated Hospital of Sun Yat-Sen University, Guangzhou, China; ^3^ Department of Biochemistry, Zhongshan Medical School, Sun Yat-Sen University, Guangzhou, China

**Keywords:** lateral mesoderm, mesenchymal stem cells, human pluripotent stem cells, differentiation, Hox genes

## Abstract

Mesenchymal stem cells (MSCs) are among the most promising cell sources for the treatment of various diseases. Nonetheless, the therapeutic efficacy in clinical trials has been inconsistent due to the heterogeneity of MSCs, which may be partially attributed to their undefined developmental origins. The lateral mesoderm is also a developmental source of MSCs that constitute appendicular skeletal elements in the developing vertebrate embryo. However, it is difficult to isolate homogeneous lateral mesoderm (LM)-derived MSCs from bone tissues or bone marrow due to the lack of understanding of their characteristics. Herein, we successfully established an efficient differentiation protocol for the derivation of MSCs with a LM origin from human pluripotent stem cells (hPSCs) under specific conditions. LM-MSCs resembled bone marrow-derived MSCs (BMSCs) with regard to cell surface markers, global gene profiles, and immunoregulatory activity and showed a homeodomain transcription factor (HOX) gene expression pattern typical of skeletal MSCs in long bones. Moreover, we demonstrated that LM-MSCs had an increased osteogenic/chondrogenic differentiation capacity and hematopoietic support potential compared to BMSCs. These homogeneous LM-MSCs may serve as a powerful tool for elucidating their precise role in bone formation and hematopoiesis and could be a potentially ideal cell source for therapeutic applications.

## Introduction

Mesenchymal stem cells (MSCs) are present in various organs and tissues, including bone marrow, adipose tissue, and the umbilical cord (Jiang et al., 2019). Due to their multilineage differentiation, immunomodulatory profiles, and hematopoietic support capabilities, MSCs have become one of the most promising sources in cell replacement therapy for the treatment of different diseases (Kassem et al., 2004; Augello and De Bari, 2010). According to ClinicalTrials.gov, the number of MSC-related clinical trials has expanded consistently since 2006, and more than 1300 MSC clinical trials have been registered to date for evaluating the treatment of neurological, joint, and graft-versus-host diseases. However, the translation of MSCs to the clinic is still progressing slowly (Herman et al., 1988; Kabat et al., 2020). The major clinical challenges with MSC therapies include the inconsistent therapeutic efficacy caused by the functional heterogeneity of MSCs (Costa et al., 2021; Mabuchi et al., 2021), which may be due to variations in donors, tissue types, and even the isolation protocols and the number of passages of MSCs (Kern et al., 2006; Chen et al., 2015; Yin et al., 2019; Costa et al., 2021).

MSCs also have multiple developmental origins, including the neural crest (neural ectoderm), mesoderm, and trophoblast. For example, craniofacial bones are generated by neural crest-derived MSCs, but the axial and appendicular bones are derived from paraxial and lateral mesoderm (LM)-derived MSCs, respectively (Olsen et al., 2000; Isern et al., 2014). Furthermore, maxilla-derived MSCs are negative for homeodomain transcription factor (HOX) gene expression, while ilium-derived MSCs highly express HOX genes and exhibit superior differentiation to chondrocytes compared with maxilla- and mandible-derived MSCs (Matsubara et al., 2005; Onizuka et al., 2020), further suggesting that MSCs of different origins possess variable functions. The above evidence indicates that the functional heterogeneity of MSCs may be partially due to their undefined developmental origins.

The LM forms the progenitor cells that constitute the heart and cardiovascular system, blood, and appendicular skeletal elements (bone and cartilage) in the developing vertebrate embryo (Prummel et al., 2020). Previous studies have shown that LM-derived MSCs (LM-MSCs) exist in the bone marrow of long bones (Isern et al., 2014). However, the lack of understanding of their characteristics (e.g., specific surface markers) makes it critically challenging to discriminate and isolate MSCs of LM origin directly from human tissues. In recent years, human pluripotent stem cells (hPSCs), including human embryonic stem cells (hESCs) and human induced pluripotent stem cells (hiPSCs), have shown promise for regenerative medicine. hPSCs can indefinitely proliferate and differentiate into almost all cell types of the three germ layers *in vitro*, including nerve cells, skeletal muscle, kidney cells, cardiomyocytes, and hepatocytes (Loh et al., 2016). More recently, studies have demonstrated that MSCs can be obtained from hPSCs through different intermediate stages, including the neural crest, neuromesoderm, mesoderm, and trophoblast (Wang et al., 2016; Chijimatsu et al., 2017; Slukvin and Kumar, 2018; Wang et al., 2019). Accordingly, we assumed that it would be possible to generate homogeneous MSCs through the intermediate stage of LM from hPSCs, which would be valuable in elucidating their distinct biological characteristics and may help to minimize the heterogeneity and variability of treatment effects in the clinical translation of MSCs.

Herein, we successfully established an efficient differentiation protocol for the derivation of MSCs with LM origin from hPSCs. LM-MSCs derived from hPSCs expressed traditional MSC surface markers and HOX genes typical of skeletal MSCs in long bones. LM-MSCs also shared similar immunoregulatory activity as bone marrow-derived MSCs (BMSCs). Moreover, we found that LM-MSCs showed an increased capacity for osteogenic and chondrogenic differentiation and hematopoietic support potential compared to BMSCs.

## Materials and Methods

### Cell Culture

We used the hESC lines H1 and H9 (Thomson et al., 1998) and the hiPSC lines HEF-hiPSCs and HDF-hiPSCs, which were established in our laboratory (Li et al., 2018), for MSC differentiation. Undifferentiated hPSCs were cultured on Matrigel (BD Biosciences, San Diego, CA, United States)-coated plates in mTeSR1 medium (StemCell Technologies, Vancouver, Canada). The medium was changed daily, and the cells were passaged every 3–4 days using ReLeSR™ (StemCell Technologies). Human BMSCs were used as a control (Chen et al., 2019). BMSCs were maintained in StemFit medium, a chemically defined medium (Ajinomoto, Tokyo, Japan), and passaged using StemPro Accutase Cell Dissociation Reagent (Life Technologies, Carlsbad, CA, United States) when the cell confluence reached 80–90%.

### Differentiation of Human Pluripotent Stem Cells Into Lateral Mesoderm-Mesenchymal Stem Cells

For induction of differentiation, confluent hPSCs were digested into single cells by incubation in Accutase at 37°C for 2–3 min. These cells were seeded onto Matrigel-coated plates at a density of 1–2×10^4^ cells/cm^2^ and cultured in mTeSR medium containing 10 μM Y27632 (ROCK inhibitor; Sigma-Aldrich, St. Louis, MO, United States) for 24 h. Next, the cells were incubated in Stage 1 medium, which consisted of DMEM/F12, 1% (v/v) penicillin/streptomycin, 1% (v/v) nonessential amino acids (NEAAs), 1% (v/v) insulin-transferrin-selenium (ITS), 1% (v/v) l-glutamic acid (L-GLU) (all from Gibco, Grand Island, NY, United States), and 3 μM CHIR99021 (Sigma-Aldrich), for an additional 48 h to differentiate into primitive streak (PS) cells. PS cells were differentiated into LM by Stage 2 medium with DMEM/F12, 1% (v/v) penicillin/streptomycin, 1% (v/v) NEAA, 1% (v/v) ITS, 1% (v/v) L-GLU, 3 μM CHIR99021, and 100 ng/ml bone morphogenetic protein-7 (BMP-7) (Peprotech, Rocky Hill, New Jersey, United States) for another 6 days. For Stage 3 MSC differentiation, LM cells were cultured in StemFit MSC medium (Ajinomoto, Tokyo, Japan) for 3–4 weeks. Accutase was used for cell passaging when differentiated cells reached subconfluence. Gene expression levels of markers typical of pluripotency, primitive streak, ectoderm, mesoderm, or endoderm were analyzed by quantitative real-time PCR (qRT-PCR) and immunostaining during each stage of differentiation. The phenotype and multipotency of LM-derived MSCs (LM-MSCs) were assessed by flow cytometry (FCM) analysis and multilineage (osteogenic, adipogenic, and chondrogenic) differentiation assays, respectively.

### Flow Cytometry Analysis

FCM was used to detect the protein expression of the intranuclear transcription factors HAND1 and FOXF1 in LM cells, surface antigens (including CD29, CD44, CD73, CD90, CD105, CD166, CD45, CD34, CD11b, CD19, and HLADR) in LM-MSCs, and CD34 in *vitro* expanded hematopoietic stem cells (all from BD Biosciences, Palo Alto, CA, United States) (Supplementary Table S1). An irrelevant isotype-identical antibody (BD Biosciences) was used as a negative control. The cells were digested by Accutase, filtered, and incubated with a special human monoclonal antibody. FCM was performed using a Beckman Coulter flow cytometer, and the data were analyzed with FlowJo software (BD Biosciences) or CytExpert (Beckman Coulter GmbH, Krefeld, Germany).

### Multilineage Differentiation of Lateral Mesoderm-Mesenchymal Stem Cells

For osteogenic differentiation, LM-MSCs were seeded at 100,000 cells/well in 6-well plates and cultured with osteogenic medium containing 0.1 mM dexamethasone, 50 mM ascorbate-2-phosphate, 10 mM b-glycerophosphate (all from Sigma-Aldrich), and 1% (v/v) penicillin/streptomycin (Gibco) for 21 days. The medium was replaced every 3 days. After 21 days, the osteogenic differentiation ability of LM-MSCs was evaluated by qRT-PCR for osteoblast-specific genes and Alizarin Red S staining to detect calcium deposits. Fifteen minutes after Alizarin Red S staining, the excess Alizarin Red solution was removed. Cells were washed 4 times with PBS and photographed with a phase-contrast microscope. Then, 10% cetylpyridinium solution was added to dissolve the color for 15 min, and the staining intensity was quantified by measuring the absorbance at 562 nm by a microplate reader (Tecan Trading AG, Switzerland).

For chondrogenic differentiation, a pellet culture system was applied. In brief, LM-MSCs were seeded at 500,000 cells/pellet in a 15 ml centrifuge tube and cultured with ACF chondrogenic medium (Stem Cell Technologies) for 21 days. Subsequently, the chondrogenic differentiation ability of LM-MSCs was evaluated by qRT-PCR analysis for chondrocyte-specific genes and Alcian blue staining to detect acid mucins. ImageJ software (National Institutes of Health) was used to analyze the diameter of cartilage micromass.

For the adipogenic differentiation assay, LM-MSCs were seeded at 100,000 cells/well in 6-well plates and cultured with adipogenic medium, which contained 0.5 mM isobutyl-methylxanthine (Sigma-Aldrich), 1 mM dexamethasone, 10 mM insulin (Sigma-Aldrich), 200 mM indomethacin (Sigma-Aldrich), and 1% (v/v) penicillin/streptomycin (Gibco), for 3 weeks. The medium was replaced every other day. The adipogenic differentiation ability of LM-MSCs was evaluated by qRT-PCR analysis for adipocyte-specific genes and oil red O staining to detect lipid droplets. After removal of the dye solution, the cells were washed three times with PBS and photographed with a phase-contrast microscope. Then, the cells were incubated with 100% isopropanol for 15 min to extract the oil red O dye from the stained lipid droplets. The OD values were measured at 510 nm by a microplate reader.

### Cell Counting Kit-8 Assay

The cell proliferation of LM-MSCs was analyzed using the fluorescence-based Cell Counting Kit-8 (CCK8) Cell Proliferation Assay (Dojindo, Kumamoto, Japan). The cells were dissociated using Accutase and seeded onto a 96-well plate at 1,500 cells/well. After 24 h, 100 μl of fresh culture medium containing 10% CCK8 solution was added to each well, and the plate was incubated for 2 h. Cell proliferation was assessed by measuring absorbance at 450 nm using a microplate reader (Tecan Trading AG, Switzerland) daily until the cells reached confluence.

### *In vitro* Immunoregulatory Activity of Lateral Mesoderm-Mesenchymal Stem Cells

For the lymphocyte proliferation assay, blood samples were collected from healthy donors after receiving informed consent. Peripheral blood mononuclear cells (PBMCs) were enriched using standardized density gradient techniques. The CD3^+^ T lymphocytes were isolated from PBMCs by FCM and then stained with 5 μm carboxylated fluorescein succinimide (CFSE; Life Technologies) according to the manufacturer’s instructions. The proliferation of labeled CD3^+^ T cells that were cocultured with or without LM-MSCs was stimulated with anti-CD3 and anti-CD28 (both from BD Biosciences) for 96 h and evaluated by FCM analysis.

For analysis of CD3^+^ T-cell intracellular cytokine production, CD3^+^ T cells were cultured with or without MSCs for 72 h. Then, 10 ng/ml brefeldin A (BFA), 50 ng/ml phorbol-12-myristate-13-acetate (PMA), and 1 μg/ml ionomycin (all from Sigma-Aldrich) were added to the culture medium for the last 6 h. Next, CD3^+^ cells were fixed, permeabilized, and incubated with antibodies against CD3, and intracellular cytokines were detected with anti-human IFN-γ-PECY7 and anti-TNF-α-PE antibodies by FCM.

For analysis of intracellular cytokine production in human THP-1-derived macrophages, THP-1 cells were seeded at 2,000,000 cells/well in 6-well plates for macrophage induction using 100 ng/ml PMA on the first day. BMSCs and LM-MSCs were preseeded onto a 24-well plate at a density of nearly 100%. On Day 2, macrophages were digested and cocultured with BMSCs or LM-MSCs at a density of 200,000 cells/well for 24 h. On Day 3, 1 ng/ml LPS and 20 ng/ml IFN-γ were added to the coculture system for another 24 h. On Day 4, the macrophages were stimulated with 10 μg/ml BFA for 6 h before FCM. Then, the cells were collected, and the expression of TNF-α in CD45 ^+^ macrophages was detected by FCM.

For the pro-inflammatory cytokine stimulation assay, 20 ng/ml IFN-γ was added to the culture medium when LM-MSCs reached 60% confluence, and the cells were cultured for 24 or 48 h. Next, the cells were collected, and the mRNA transcripts of pro-inflammatory mediators (IL6, IL8, and CCL2) and anti-inflammatory mediators (IDO, PDL1, and TSG6) were analyzed by qRT-PCR.

### *In vitro* Culture of Hematopoietic Stem Cells With Lateral Mesoderm-Mesenchymal Stem Cells

Human umbilical cord blood CD34 ^+^ hematopoietic stem cells were collected from healthy donors following the Declaration of Helsinki protocols with fully ethical informed consent and enriched with FCM. BMSCs and LM-MSCs were preseeded onto a 24-well plate at a density of 90%. On the second day, BMSCs and LM-MSCs were treated with mitomycin c (0.5 mg/ml) for 3 h and washed with PBS 3 times. Then, CD34 ^+^ HSCs (5,000 cells) were inoculated into each well of 24-well plates and cocultured with BMSCs or LM-MSCs in StemSpan^Tm^ CD34 ^+^ Expansion medium (Stem Cell Technologies) for 9 days. The CD34 ^+^ HSCs cultured alone in the same media acted as controls. After 9 days of culture, FCM was used to detect the maintenance of CD34 ^+^ HSCs in different coculture conditions.

### Long-Term Culture Initiating Cell Assays

BMSCs and LM-MSCs were treated with mitomycin c as described above. Then, CD34 ^+^ HSCs (1,000 cells) were inoculated onto each well of 24-well plates and cocultured with BMSCs or LM-MSCs in StemSpan^Tm^ CD34 ^+^ Expansion medium (Stem Cell Technologies). After culture for 35 days, 10,000 cells isolated from the supernatant of the coculture system were seeded in methylcellulose (Stem Cell Technologies) plates and incubated at 37°C in 5% CO_2_. Fourteen days later, the colonies that were formed were counted and analyzed.

### Tumor Formation Assay

For evaluation of the potential tumor formation risk of hPSC-derived LM-MSCs, 5 ×10^6^ cells were premixed with 100 μl of PBS containing 30% Matrigel and injected subcutaneously into 6-week-old male NCG mice (n = 5 for each cell line; obtained from Vital River, Beijing, China). BMSCs and undifferentiated hPSCs were used as controls. Tumor occurrence was evaluated 8 weeks after cell injection. All experimental procedures involving animals were approved by the Animal Ethics Committee of Sun Yat-Sen University.

### *In vivo* Bone Formation Assay

For evaluation of the *in vivo* bone formation capacity of LM-MSCs, we used hydroxyl-apatite/tricalcium phosphate ceramic powder (HA/TCP; Zimmer Scandinavia, Denmark) assembled with LM-MSCs and transplanted the cell-scaffold complexes subcutaneously onto the dorsal surfaces of 8-week-old male NCG mice (n = 5 for each group), as previously described (Wang et al., 2019). The subcutaneous grafts were collected 8 weeks after transplantation, decalcified in 10% ethylenediaminetetraacetic acid (EDTA) for 3 weeks and embedded in paraffin. For microstructure observation, 4-µm-thick sections of the grafts were analyzed by Masson’s trichrome staining, hematoxylin and eosin (H&E) staining, and immunostaining for the detection of osteogenic-related markers and hematopoietic-related markers. All experimental procedures involving animals were approved by the Animal Ethics Committee of Sun Yat-Sen University.

### Quantitative Reverse Transcription-Polymerase Chain Reaction

Total RNA was extracted using RNAzol (Molecular Research Center, Cincinnati, OH, United States) according to the manufacturer’s protocol and then reverse transcribed into cDNA using a Quantitect Reverse Transcription kit (Qiagen, Valencia, CA, United States) in a 10 μl reaction system. qRT-PCR was performed using a DyNAmo ColorFlash SYBR Green qPCR kit (Thermo Fisher Scientific, Rutherford, NJ, United States) on a LightCycler 480 Detection System (Roche Diagnostics, Mannheim, Germany). The thermocycling conditions consisted of 40 cycles of denaturation at 95°C for 10 s, annealing at 60°C for 20 s, and extension at 72°C for 30 s. GAPDH was used as an internal control, and gene expression levels were calculated using the 2-ΔΔCT method. The primer sequences are presented in Supplementary Table S2.

### Western Blot

Cells or tissues were digested, washed with cold PBS, and lysed in 1x RIPA buffer containing phenylmethanesulfonyl fluoride (PMSF) on ice to extract protein. After sonication, the proteins were separated by SDS-PAGE and electrically transferred to a polyvinylidene difluoride membrane with a pore size of 0.45 μm (Millipore, Bedford, MA, United States). Each membrane was blocked with 5% BSA solution and incubated with appropriate primary antibodies diluted with TBS/T containing 1% BSA overnight at 4°C, followed by reaction with horseradish peroxidase (HRP)-linked secondary antibodies (Cell Signaling Technology, Beverly, MA, United States) at room temperature for 1 h before chemiluminescence detection. The antibodies used in western blotting are listed in Supplementary Table S3.

### Immunocytochemistry

For immunocytochemistry, differentiated cells were fixed with 4% paraformaldehyde for 20 min, blocked with blocking buffer (0.2% Triton X-100 with 5% human BSA) for 1 h, and incubated with primary antibody (appropriate antibody concentration with 5% human BSA) at 4°C overnight. The cells were washed three times with PBS for 5 min and then incubated at room temperature for 1 h with the secondary antibody in the dark. The nucleus was stained with 4′,6-diamino-2-phenylindole (DAPI; Sigma-Aldrich), and the results were photographed and analyzed by fluorescence microscopy. The utilized antibodies for immunocytochemistry are listed in Supplementary Table S3.

### RNA Sequencing

Samples for RNA sequencing included hiPSC-derived LM cells (LM 1, LM 2), hiPSC-derived LM-MSCs (LM-MSCs 1, LM-MSCs 2), and BMSCs (BMSCs 1, BMSCs 2). Total mRNA was extracted and isolated by RNAzol (Molecular Research Center), and RNA sequencing libraries were constructed using an Illumina mRNA-seq Prep Kit (Illumina, San Diego, CA, United States) as recommended by the manufacturer. The fragmented and randomly primed 150 bp paired-end libraries were sequenced using the Illumina HiSeq X Ten platform. Sequencing data were processed using Consensus Assessment of Sequence and Variation (CASAVA, version 1.8.2; Illumina) under the default settings. The reads per kilobase of transcript per million mapped reads (RPKM) values were used to evaluate the expression levels of genes, and Pearson’s correlations were calculated (*R* ^2^) to measure the similarities between different cell lines. The RNA-Seq data were also analyzed using Ingenuity Pathways Analysis (IPA) software (Ingenuity Systems, Inc., Redwood City, CA, United States) to categorize the differentially regulated genes. RNA-seq data were deposited in the Gene Expression Omnibus (GEO) under the accession number GSE182161.

### Statistical Analysis

All experiments shown were biologically replicated at least three times. All results are shown as the mean ± standard deviation (SD) from at least three independent experiments. Differences between groups were tested by independent sample *t* test and one-way analysis of variance (ANOVA) with a post hoc test using the Student–Newman–Keuls test. Correlation analysis was used to measure the similarities between the different lines. All data were analyzed by GraphPad Prism 7 software and are presented as the mean plus standard deviation. ∗*p* < 0.05, ∗∗*p* < 0.01, and ∗∗∗*p* < 0.001 were considered statistically significant.

## Results

### Differentiation of Human Pluripotent Stem Cells Into Primitive Streak Cells Using CHIR99021

**FIGURE 1:**
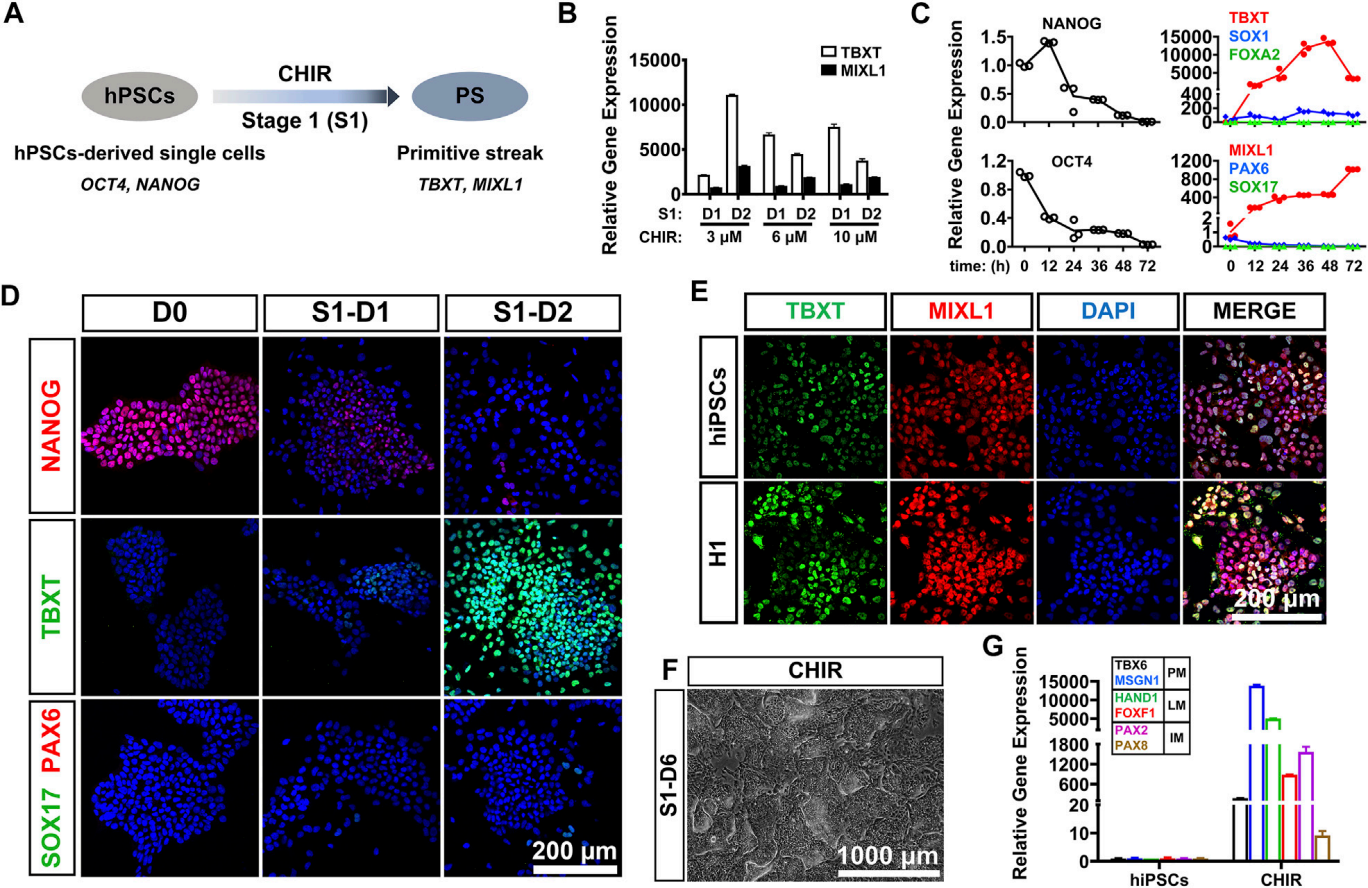
CHIR99021 efficiently induced primitive streak differentiation from hPSCs. **(A)**. Diagram of primitive streak (TBXT; MIXL1) differentiation from hPSCs (OCT4; NANOG) using CHIR99021 (CHIR) at stage 1 (S1). **(B)**. qRT-PCR detection of the expression of primitive streak (PS) markers (TBXT and MIXL1) when hiPSCs were treated with different concentrations (3, 6, and 10 μM) of CHIR for 1 day (D1) and 2 days (D2) at stage 1. **(C)**. Time course of gene expression in the hiPSCs treated with 3 μM CHIR for 72 h at stage 1. qRT-PCR detection of the gene expression levels of markers for pluripotency (OCT4; NANOG), ectoderm (SOX1; PAX6), primitive streak (TBXT; MIXL1), and definitive endoderm (SOX17; FOXA2). **(D)**. Immunostaining assay for the markers of pluripotency (NANOG), primitive streak (TBXT), definitive endoderm (SOX17), and ectoderm (PAX6) in the hiPSCs treated with 3 μM CHIR at Day 0 (D0), Day 1 (S1-D1), and Day 2 (S1-D2). Scale bar: 200 μm. **(E)**. Immunofluorescence staining for the expression of the PS markers TBXT and MIXL1 in the hiPSCs and H1 hESCs treated with CHIR for 2 days. Scale bar: 200 μm. **(F)**. Phase-contrast image of the hiPSCs treated with CHIR for 6 days (S1-D6). Scale bar: 1,000 μm. **(G)**. qRT-PCR detection of the gene expression of markers for paraxial mesoderm (TBX6; MSGN1), lateral mesoderm (HAND1; FOXF1), and intermediate mesoderm (PAX2; PAX8) when hiPSCs were treated with basal medium (basal) or 3 μM CHIR for 6 days. Undifferentiated hiPSCs were used as a negative control.

PS cells serve as a conduit for the generation of mesoderm and definitive endoderm during early embryogenesis (Rivera-Perez and Magnuson, 2005). For development of an induction protocol for LM, it is critical to develop an efficient method to differentiate hPSCs into PS cells, which coexpress BRACHYURY (TBXT) and MIXL1 (Figure 1A). Previous studies have shown that treatment with a certain concentration of CHIR99021 (CHIR; a glycogen synthase kinase-3 inhibitor and WNT activator) for 24 or 48 h could effectively induce hPSCs to differentiate into PS cells (Lam et al., 2014; Liu et al., 2020). In this study, we first investigated the differentiation kinetics of the CHIR-treated hPSCs (at 3–10 μM) during PS induction (stage 1 differentiation). Phase-contrast images showed similar morphological changes when hiPSCs were treated with different concentrations of CHIR during PS differentiation (Supplementary Figure S1A). qRT-PCR analysis revealed that the mRNA level of TBXT, a PS-specific marker, was rapidly upregulated (5,000–10,000-fold) in all the CHIR-treated cells compared to the undifferentiated hiPSCs 24 h after treatment. However, the TBXT expression in the 6 and 10 μM CHIR-treated cells started to decrease 48 h later, while continuously increased mRNA expression of TBXT (up to 15,000-fold) was observed after treatment with 3 μM CHIR for 2 days. The mRNA expression of MIXL1, the other PS marker, was also upregulated significantly upon CHIR treatment, with the highest level observed with 3 μM CHIR (Figure 1B). These results indicated that a CHIR concentration of 3 μM could yield higher expression of TBXT/MIXL1 in differentiated hiPSCs than the other concentrations tested and was then utilized for the subsequent differentiation study.

We then tried to determine the optimal treatment time of CHIR and detected the gene expression patterns of the 3 μM CHIR-treated cells during the 72-h differentiation process. We found that the expression of pluripotency genes (OCT4, NANOG) was downregulated sharply, while the mRNA levels of genes related to ectoderm (PAX6, SOX1) and endoderm (SOX17, FOXA2) were mildly elevated or remained unaffected after treatment with 3 μM CHIR for 2 or 3 days (Figure 1C). These data suggested that hiPSCs quickly lost their undifferentiated state, and minimal ectodermal or endodermal progenies were generated in our differentiation system. More importantly, we found that prolonged treatment with CHIR over 48 h resulted in a significant reduction in TBXT expression in hiPSCs, although a continuously increased expression level of MIXL1 was noted (Figure 1C). Therefore, we decided to employ a 2-days differentiation protocol for primitive streak commitment from hiPSCs. We performed immunostaining analysis and found that the protein expression of TBXT increased in a time-dependent manner, and almost all of the differentiated cells were TBXT positive after the addition of 3 μM CHIR for 2 days. These results also showed that conditioning with CHIR led to the gradually diminished expression of the pluripotency marker NANOG, while the expression of SOX17 (endoderm progenitor marker) and PAX6 (ectoderm progenitor marker) was rarely activated in these cells during hiPSC differentiation (Figure 1D). Furthermore, we discovered that 48 h after 3 μM CHIR treatment, the gene and protein expression patterns in the hESC line (H1) were similar to those in hiPSCs, as illustrated by an immunofluorescence assay with anti-TBXT and anti-MIXL1 antibodies, and nearly 100% TBXT+/MIXL1+ cells were generated in both cell lines (Figure 1E). Together, these data indicated that treatment with 3 μm CHIR for 48 h could efficiently induce hPSCs to differentiate into primitive streak cells.

### Generation of Lateral Mesoderm From the Primitive Streak by Coactivation of the WNT and Bone Morphogenetic Proteins Signaling Pathways

It was reported that exposure to CHIR resulted in hPSC differentiation toward an LM fate (Lam et al., 2014). To test whether treatment with CHIR alone could efficiently induce LM differentiation, we cultured hiPSCs in medium containing 3 μM CHIR for 6 days. We found that heterogeneous cell populations with diverse cellular morphologies were generated by prolonged treatment with CHIR (Figure 1F). qRT-PCR revealed that the mRNA levels of markers for paraxial mesoderm (PM; TBX6/MSGN1), intermediate mesoderm (IM; PAX2/PAX8) and LM (HAND1/FOXF1) were all markedly upregulated in differentiated cells when compared to undifferentiated hiPSCs (Figure 1G). Immunofluorescence assays also showed that only 40–50% of cells were HAND1-positive (Supplementary Figure S1B). These data suggested that CHIR alone was not sufficient to induce the formation of LM.

**FIGURE 2:**
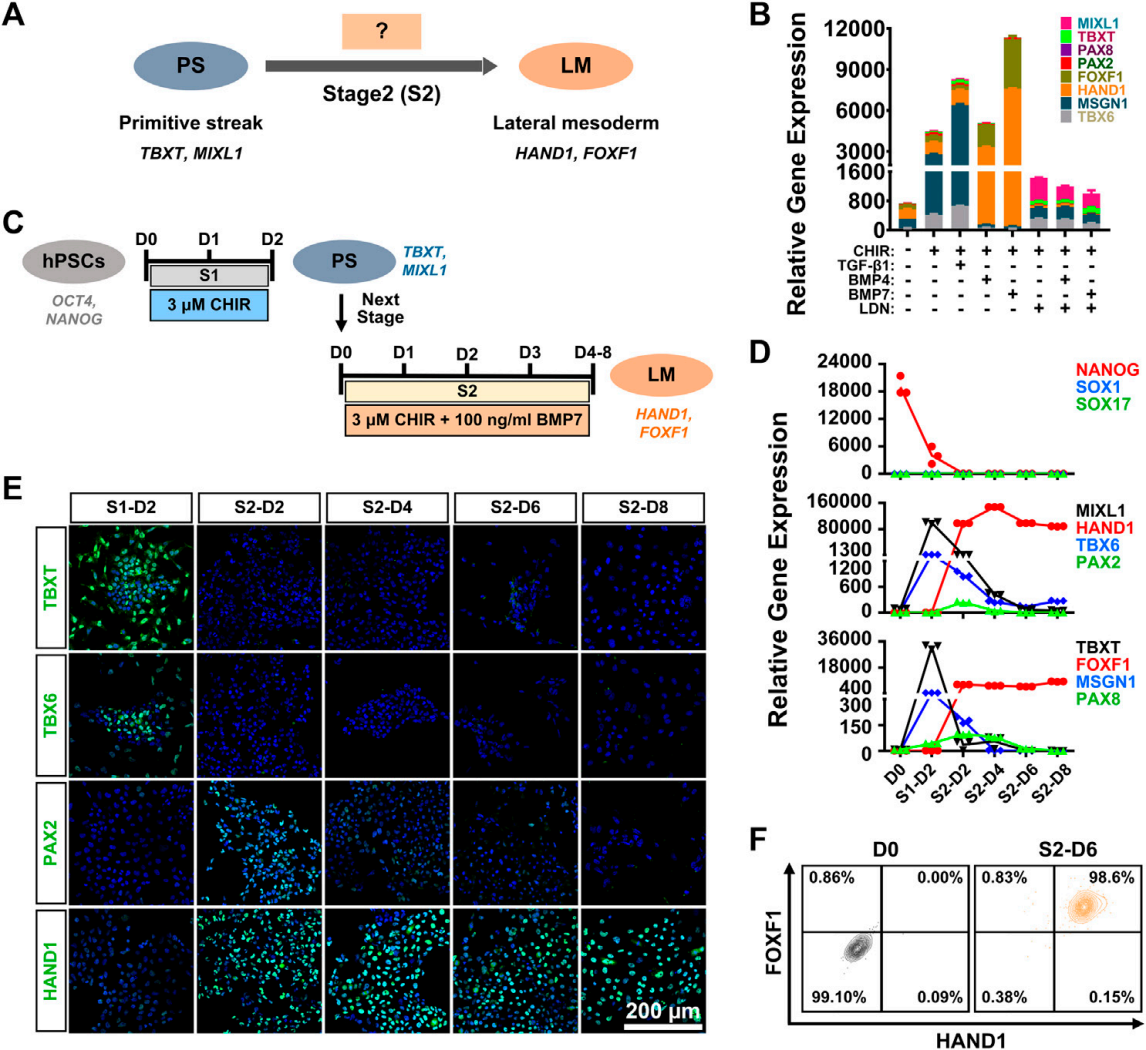
Generation of lateral mesoderm from the primitive streak by activation of the WNT and BMP signaling pathways. **(A)**. Diagram of lateral mesoderm (HAND1; FOXF1) differentiation from the hPSC-derived primitive streak (TBXT; MIXL1) by activating TGFβ1 or BMP signaling at stage 2 (S2). **(B)**. qRT-PCR detection of the gene expression of markers for paraxial mesoderm (TBX6; MSGN1), lateral mesoderm (HAND1; FOXF1), and intermediate mesoderm (PAX2; PAX8) when primitive streak cells were treated with different factors (CHIR, TGFβ1, BMP4, BMP7, or LDN192189) at stage 2 for 4 days (S2-D4). **(C)**. Diagram of the strategy for derivation of lateral mesoderm from hPSCs through the primitive streak with 3 μM CHIR99021 and 100 ng/ml BMP7 for 4–8 days at stage 2 (S2-D4–S2-D8). **(D)**. qRT-PCR detection of the time course of gene expression in markers for pluripotency (NANOG), ectoderm (SOX1), definitive endoderm (SOX17), the primitive streak (TBXT; MIXL1), paraxial mesoderm (TBX6; MSGN1), lateral mesoderm (HAND1; FOXF1), and intermediate mesoderm (PAX2; PAX8) when hiPSCs were treated with 3 μM CHIR99021 at stage 1 (S1) for 2 days, followed by 3 μM CHIR99021 and 100 ng/ml BMP7 at stage 2 (S2) for 8 days. **(E)**. Immunostaining for the markers of the primitive streak (TBXT), paraxial mesoderm (TBX6), lateral mesoderm (HAND1) and intermediate mesoderm (PAX2) during lateral mesoderm differentiation from the hiPSC-derived primitive streak. Scale bar: 200 μm. **(F)**. Quantification of hiPSC-derived lateral mesoderm (HADN1+/FOXF1+) cells at stage 2 for 6 days (S2-D6) by FCM.

Previous studies have demonstrated that TGF-β family-mediated SMAD signaling is involved in mesoderm specification (Schier and Shen, 2000; Casari et al., 2014), and bone morphogenetic proteins (BMPs), such as BMP4 and BMP7, play essential roles in mesoderm formation during embryogenesis (Kim et al., 2019). In particular, high levels of BMP signaling are necessary and sufficient to promote the formation of LM both *in vivo* and *in vitro* (James and Schultheiss, 2005). We attempted to verify the specificity of BMP or TGF-β signaling in LM induction from PS cells (stage 2 differentiation) (Figure 2A). hiPSC-derived PS cells were cultured in induction medium with different signaling molecules, such as BMP agonist (BMP4 or BMP7), SMAD1/5/8 inhibitor (LDN193189), or a TGF-β agonist (TGF-β1), in the presence of CHIR for 4 days. We found that supplementation with BMP or TGF-β1 could result in rapid cell expansion and relatively morphologically homogeneous cell cultures compared to CHIR treatment alone (Supplementary Figure S2A). qRT-PCR analysis showed that higher expression of LM markers (HAND1; FOXF1) and relatively lower mRNA levels of the PM markers (TBX6; MSGN1) and IM markers (PAX2; PAX8) could be detected in the BMP groups compared to the TGF-β1-, LDN193189-, or CHIR-treated or nontreated groups (Figure 2B). Moreover, we found that BMP7 displayed better efficacy in promoting LM commitment than BMP4 (Figure 2B). When the BMP pathway was inhibited by LDN193189, these cells proliferated slowly and showed obvious morphological heterogeneity (Supplementary Figure S2B), and the expression levels of the detected mesoderm-related genes (MSGN1, HAND1) were downregulated significantly compared to those in the TGF-β1 or BMP group (Figure 2B). These results suggested that the BMP signaling pathway, in particular BMP7, was critically involved in the determination of LM fate from the PS *in vitro*. Indeed, western blotting analysis showed that treated with both CHIR and BMP7 (CHIR + BMP7) significantly activated the BMP signaling pathway by inducing SMAD1/5/8 phosphorylation, while “CHIR + TGFβ1” group exhibited stronger level of phosphorylated SMAD2/3 (Supplementary Figure S2B). These results further indicated that the activation of BMP-SMAD1/5/8 signaling plays an important role in PS cell-specific induction of LM.

To further determine the optimal induction time for LM differentiation from the PS, we first induced hiPSCs to differentiate into the PS with 3 μM CHIR for 2 days (stage 1; S1) and then cultured them in the presence of both CHIR (3 μM) and BMP7 (100 ng/ml) (stage 2; S2) for 4–8 days (stage 2; Figure 2C). A relatively homogeneous cell population could be obtained after LM induction (Supplementary Figure S2C). qRT-PCR revealed that the expression of NANOG decreased rapidly on day two at stage 1 differentiation (S1-D2) and showed extremely low levels during the subsequent differentiation process, while genes indicative of ectoderm (SOX1) and endoderm (SOX17) were minimally expressed throughout the differentiation period. The expression of the PS cells (TBXT/MIXL1) reached a peak on S1-D2 and decreased quickly when the medium was switched to stage 2 induction medium. The qRT-PCR results also indicated that PS cells started to differentiate into mesodermal cells, as evidenced by elevated expression of the markers of PM (TBX6/MSGN1), LM (HAND1/FOXF1), and IM (PAX2/PAX8) since S1-D2 or S2-D2. Nonetheless, transcript levels of PM- or IM-specific genes were significantly downregulated from S2-D4, while mRNA levels for LM markers (HAND1/FOXF1) increased constantly during the induction period and maintained a high level up to S2-D6 or S2-D8 (Figure 2D). The immunofluorescence assays showed that differentiated cells transiently expressed TBXT (S1-D2), TBX6 (S1-D2), and PAX2 (from S2-D2 to S2-D4) and then expressed only the LM marker HAND1 from S2-D6 (Figure 2E). To confirm these findings, we used flow cytometry to quantify the LM differentiation efficiency on S2-D6, showing that 99.6 ± 0.51% of the differentiated cells were HAND1 positive, and 98.6 ± 0.4% of the cells were double positive for HAND1 and FOXF1 (Figure 2F). More importantly, a similar high efficiency of LM differentiation could be achieved in the H1 cell line using the same differentiation protocol, as displayed by immunostaining, qRT-PCR, and FCM (Supplementary Figure S2D–F). These data demonstrated that PS could be effectively induced into LM when treated with 3 μM CHIR and 100 ng/ml BMP7 for 6 days.

### Derivation and Characterization of Mesenchymal Stem Cells Derived From Human Pluripotent Stem Cell-Lateral Mesoderm *in vitro*

LM-derived mesenchymal cells are present throughout the anteroposterior axis of the embryo (Gros and Tabin, 2014) and are closely associated with the formation of adult appendicular skeletal elements (bone and cartilage) (Cohn and Tickle, 1996; Olsen et al., 2000) and visceral white fat (Chau et al., 2014). Such evidence suggests that an MSC-like population exists during both LM differentiation and homeostatic maintenance of LM-derived tissues (Sheng, 2015). Thus, we hypothesize that MSCs could be obtained *in vitro* from hPSCs via an intermediate stage of LM.

**FIGURE 3:**
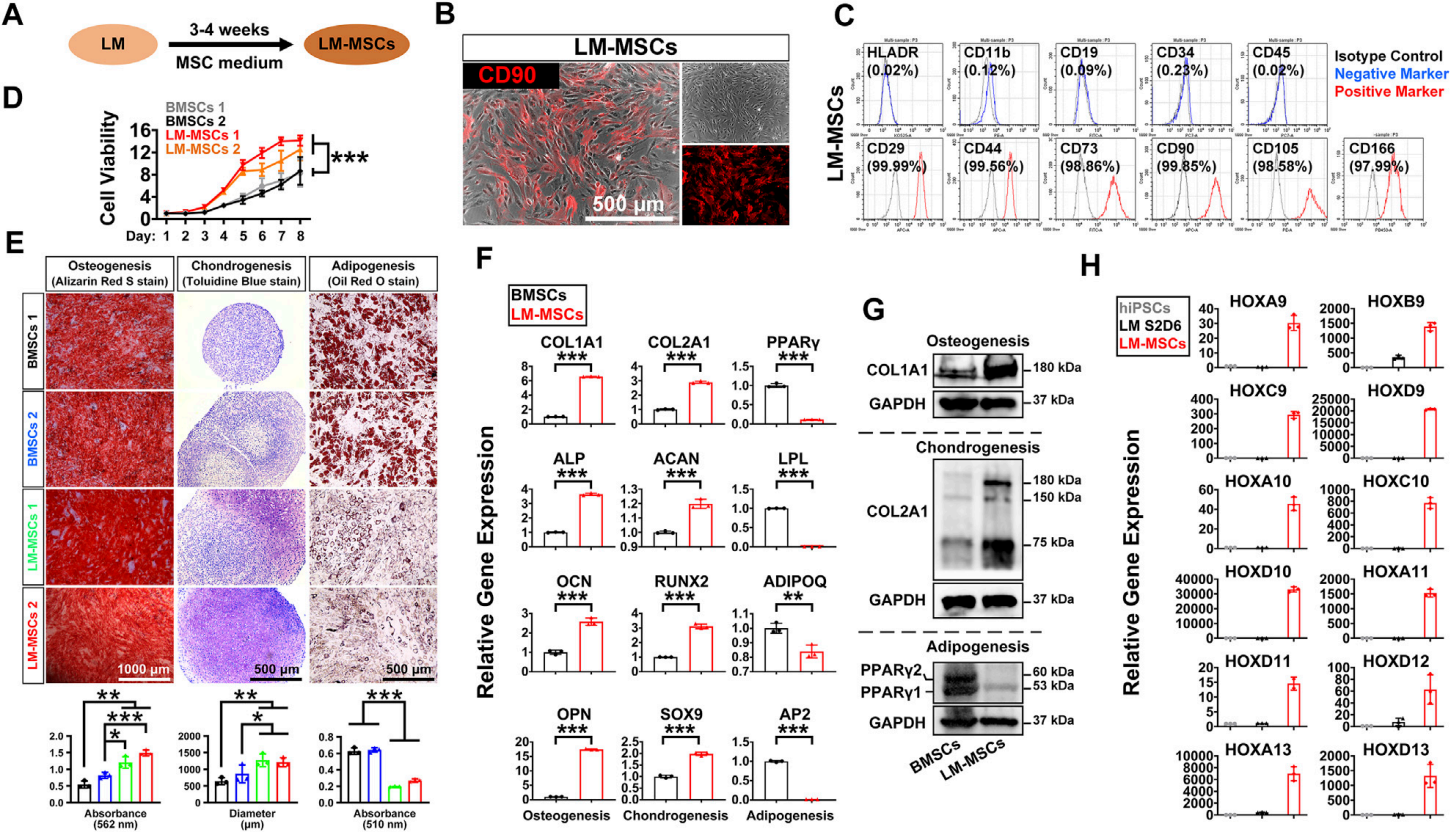
Derivation and characterization of LM-MSCs from hiPSCs. **(A)**. Diagram of the strategy for derivation of MSCs from the hPSC-derived lateral mesoderm by treatment with MSC medium for 3–4 weeks. **(B)**. Phase-contrast image of the hiPSC-derived LM-MSCs and immunostaining of MSC marker (CD90). Scale bar: 500 μm. **(C)**. Flow cytometry for detection of typical MSC surface markers in the hiPSC-derived LM-MSCs. **(D)**. The proliferation of LM-MSCs and BMSCs was detected by CCK8 assays. *p < 0.05; **p < 0.01; ***p < 0.001; ns. is nonsignificant. **(E)**. The osteogenic, chondrogenic, and adipogenic differentiation potentials of LM-MSCs and BMSCs were verified by Alizarin Red S staining (scale bar: 1,000 μm), toluidine blue staining (scale bar: 500 μm), and oil red O staining (scale bar: 500 μm) respectively. The OD values of Alizarin Red S staining (562 nm) and oil red O staining (510 nm) were measured by spectrophotometry. ImageJ software was used to analyze the toluidine blue-stained cartilage micromass diameter (n = 3). *p < 0.05; **p < 0.01; ***p < 0.001; ns. is nonsignificant. **(F)**. qRT-PCR detection of the gene expression of osteogenic (COL1A1; ALP; OCN; OPN), chondrogenic (COL2A1; ACAN; RUNX2; SOX9), and adipogenic (PPARγ; LPL; ADIPOQ; AP2) markers in LM-MSCs and BMSCs. *p < 0.05; **p < 0.01; ***p < 0.001; ns. is nonsignificant. **(G)**. Western blotting for the protein expression of osteogenic (COL1A1), chondrogenic (COL2A1), and adipogenic (PPARγ) markers in differentiated LM-MSCs. **(H)**. qRT-PCR detection of the gene expression of HOX genes in hiPSCs, hiPSC-LM, and LM-MSCs.

To induce MSC differentiation from LM, we cultured hiPSC-derived LM cells (S2-D6) for 3–4 weeks in chemically defined MSC medium, StemFit for MSC (Figure 3A). We observed significant changes in cell morphology during differentiation (Supplementary Figure S3A), and cells gradually became spindle-shaped and showed active proliferation, presenting a typical whirlpool-like alignment at a high density (Figure 3B). We first tested whether LM-derived cells satisfied the 2006 ISCT’s guidelines for MSC characterization (Dominici et al., 2006). FCM analysis showed that over 95% of the differentiated cells expressed the typical surface markers of human MSCs, including CD29, CD44, CD73, CD90, CD105, and CD166, but were negative for HLADR, CD11b, CD19, CD34, and CD45 (Figure 3C, Supplementary Figure S3B). The CCK8 assay revealed that these cells exhibited stronger proliferation than the BMSCs in StemFit medium (Figure 3D). Moreover, when induced to differentiate into osteoblasts, chondrocytes, and adipocytes *in vitro*, LM-derived cells displayed stronger osteogenic and chondrogenic differentiation but less potent adipogenic differentiation than BMSCs, as shown by Alizarin Red S staining, toluidine blue staining, and oil red O staining, respectively (Figure 3E, Supplementary Figure S3C). Indeed, qRT-PCR revealed a higher expression of osteogenic (COL1A1, ALP, OCN, OPN, SP7) and chondrogenic (COL2A1, ACAN, RUNX2, SOX9) genes, while markers of adipogenesis (PPARγ, LPL, ADIPOQ, AP2) were weakly expressed in the LM-derived cells compared to the BMSCs (Figure 3F, Supplementary Figure S3D). The western blotting results further showed more abundant COL1A1/COL2A1 during osteogenic/chondrogenic commitment and a lower protein level of PPARγ during adipogenic differentiation of LM-MSCs compared to BMSCs (Figure 3G). These results suggested that LM-derived cells resemble primary BMSCs in surface marker expression and differentiation potential, and thus these cells are referred to as LM-MSCs. Moreover, LM-MSCs could be readily generated from H1 and H9-hESC lines and HDF-hiPSCs (Supplementary Figure S4A,B).

The HOX gene family plays an essential role during vertebrate limb development, and loss-of-function mutations of some HOX genes, alone or in combination, could lead to severe developmental defects in the limb (Zakany and Duboule, 2007). Thus, we investigated whether LM-MSCs could express typical HOX family members. The qRT-PCR results revealed much more abundant expression of HOX genes 9–13 in LM-MSCs (Figure 3H) than in hiPSCs or LM, suggesting that LM-MSCs had HOX expression patterns similar to those of skeletal progenitor cells in the vertebrate limb or limb skeleton-derived BMSCs (Zakany and Duboule, 1999; Rux et al., 2016).

**FIGURE 4:**
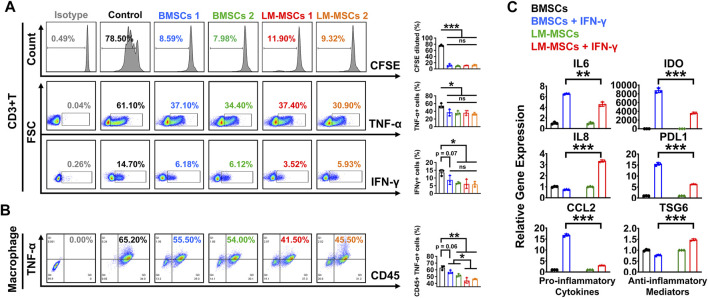
Immunomodulatory properties of LM-MSCs. **(A)**. The proliferation (CFSE assay) and production of TNF-α and IFN-γ were examined by flow cytometry when CD3^+^ T cells were cultured with or without LM-MSCs or BMSCs for 3 days. *p < 0.05; **p < 0.01; ***p < 0.001, ns. is nonsignificant. **(B)**. The production of TNF-α of human THP-1-derived macrophages was examined by flow cytometry when cells cultured with or without LM-MSCs or BMSCs for 3 days. *p < 0.05; **p < 0.01; ***p < 0.001, ns. is nonsignificant. **(C)**. qRT-PCR detection of the gene expression of pro-inflammatory cytokines (IL-6, IL-8, and CCL2) and anti-inflammatory mediators (IDO, PDL1, and TSG6) in LM-MSCs and BMSCs exposed to an inflammatory environment. *p < 0.05; **p < 0.01; ***p < 0.001; ns. is nonsignificant.

MSCs were reported to have effective immunomodulatory properties and anti-inflammatory abilities and represent a promising therapy in clinical use for a wide range of immune-related diseases (Muller et al., 2021). To explore the immunoregulatory activity of LM-MSCs, we evaluated the suppressive effects of LM-MSCs on the proliferation and pro-inflammatory cytokine production of CD3^+^ T cells. We found that when cocultured with CD3^+^ T cells, LM-MSCs exhibited an immunomodulatory function similar to that of BMSCs and could efficiently prevent the proliferation of CD3^+^ T cells and reduce the percentages of CD3^+^ T cells that produced TNF-α and IFN-γ (Figure 4A). Since macrophages play important roles in the osteogenic environment (Munoz et al., 2020), we asked whether LM-MSCs, in turn, could exert immunoregulatory effects on macrophages. The results showed that LM-MSCs inhibited the production of TNFα from human THP-1-derived macrophages more efficiently than BMSCs (Figure 4B). Previous study has revealed that the immunoregulatory function of MSCs is highly plastic. MSCs can not only inhibit the immune response but also promote it (Li et al., 2012; Wang et al., 2014). We therefore investigated the immune response of LM-MSCs when exposed to pro-inflammatory factors by qRT-PCR detection of pro-inflammatory cytokines (IL-6, IL8, and CCL2) and anti-inflammatory mediators (IDO, PDL1, and TSG6). The results indicated that IFN-γ stimulation induced the upregulation of IL-6 expression in both LM-MSCs and BMSCs (approximately 4–6-fold higher than that in the untreated cells). Interestingly, the mRNA level of IL8 was enhanced in the treated LM-MSCs but not the BMSCs, while significantly increased CCL2 transcripts (approximately 15–20-fold higher than untreated cells) were observed only in the stimulated BMSCs. We also found that the expression of anti-inflammatory cytokines was substantially augmented in both cell lines after IFN-γ treatment. Moreover, higher levels of IDO and PDL1 were detected in BMSCs, while increased TSG6 mRNA transcription was observed in LM-MSCs upon IFN-γ stimulation. These preliminary data suggested that LM-MSCs and BMSCs have similar but not identical immunomodulatory properties (Figure 4C).

### *In vivo* Transplantation of Lateral Mesoderm-Mesenchymal Stem Cells

**FIGURE 5:**
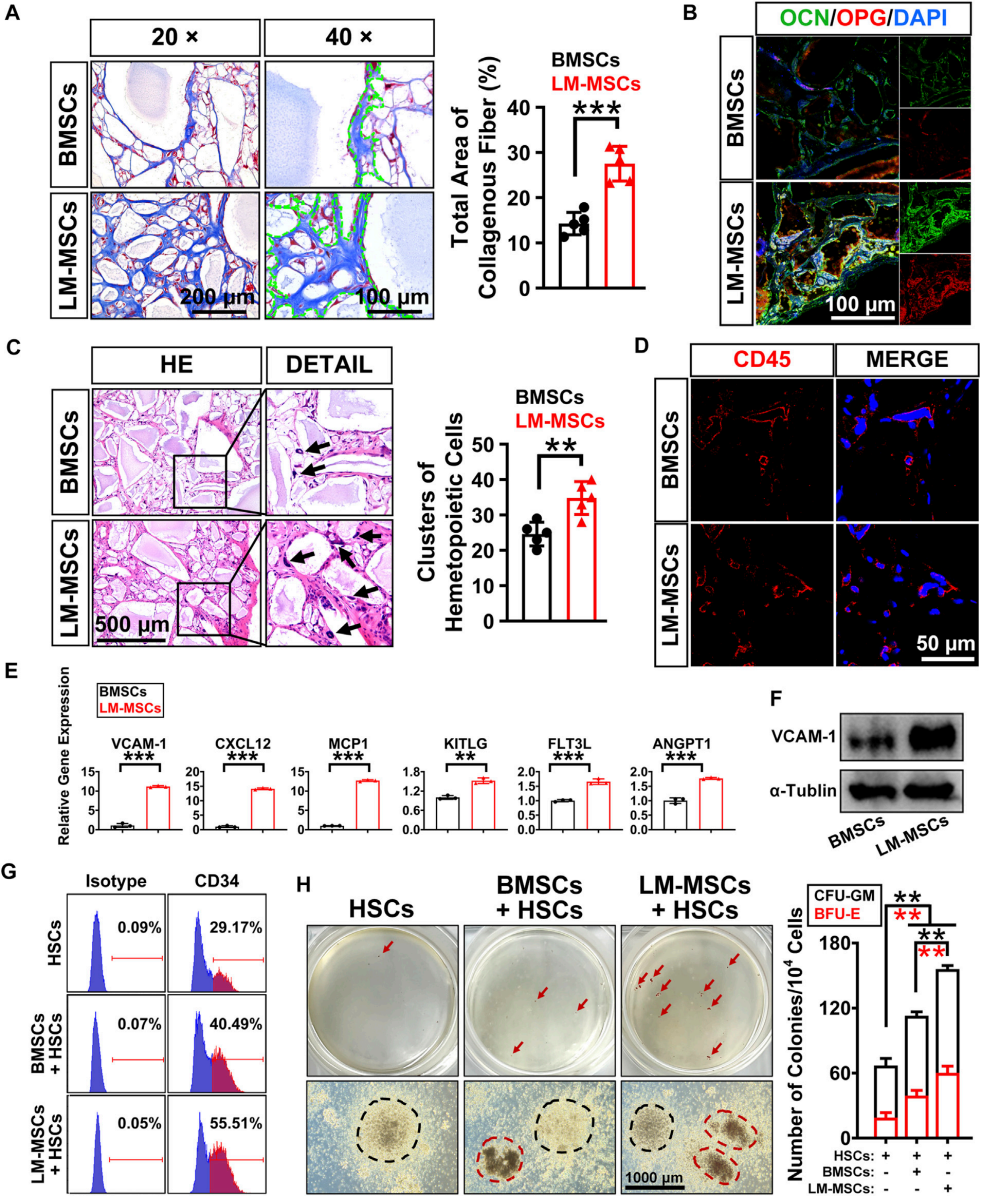
*In vivo* bone formation assay and detection of the hematopoiesis-supporting capacity of LM-MSCs. **(A)**. The samples of *in vivo* bone formation were analyzed by Masson’s trichrome staining. 20× (scale bar: 200 μm); 40× (scale bar: 100 μm). The total area percentage of collagen fibers (n = 5) was quantified by ImageJ software. **(B)**. Immunostaining of *in vivo* bone formation samples with anti-osteoprotegerin (OPG) and anti-osteocalcin (OCN) antibodies (scale bar: 100 μm). **(C)**. HE staining (scale bar: 500 μm) was performed, and the hematopoietic clusters were quantified (n = 5). *p < 0.05; **p < 0.01; ***p < 0.001; ns. is nonsignificant. **(D)**. Immunostaining with anti-mouse CD45 antibody was applied for the detection of the nucleated cells of mouse origin (scale bar: 50 μm). **(E)**. The expression of hematopoietic supporting genes in cultured LM-MSCs and BMSCs was detected by qRT-PCR. *p < 0.05; **p < 0.01; ***p < 0.001; ns. is nonsignificant. **(F)**. Western blotting for the expression of VCAM-1 in BMSCs and LM-MSCs. **(G)**. FCM analysis of the proportion of CD34 ^+^ HSCs when cocultured with LM-MSCs or BMSCs. **(H)**. The hematopoiesis-supporting function of LM-MSCs and BMSCs was compared by CFU assays. black dotted line indicates CFU-GM, while the red dotted line indicates BFU-E.

We performed subcutaneous transplantation of LM-MSCs into NCG mice to assess their *in vivo* bone formation activity. Eight weeks after transplantation of the combined LM-MSCs and hydroxyapatite scaffolds, samples were collected and analyzed (Supplementary Figure S5A). We detected enhanced bone formation (collagen deposition) in the LM-MSC group compared with the BMSC group, as revealed by Masson’s trichrome staining and the total area percentage of collagen fiber quantified by ImageJ software (Figure 5A). Immunofluorescence assays also showed that more osteoprotegerin (OPG)- and osteocalcin (OCN)-positive cells could be detected in the LM-MSC group than in the BMSC group (Figure 5B). These results were consistent with those of the *in vitro* osteogenic differentiation assay described above. More importantly, H&E staining, anti-human mitochondria and anti-mouse CD45 immunostaining revealed that more hematopoietic cell clusters/cells of mouse origin appeared in the samples derived from LM-MSCs than in those from BMSCs, indicating a better hematopoiesis-supporting capacity of LM-MSCs over BMSCs (Figures 5C,D; Supplementary Figure S5B). qRT-PCR analysis further revealed that the mRNA levels of hematopoietic stem cell maintenance genes, including CXCL12, VCAM-1, MCP1, KITLG, FLT3L, and ANGPT1, were remarkably upregulated in LM-MSCs compared to primary BMSCs (Figure 5E). Western blot results confirmed the higher protein level of VCAM-1 in LM-MSCs than in BMSCs (Figure 5F). To verify the supportive function for hematopoiesis of LM-MSCs, we performed *in vitro* coculture experiments using MSCs and HSCs. FCM analysis showed that coculture with LM-MSCs generated a significantly greater proportion of CD34 ^+^ HSCs than that of the BMSC and control groups. LM-MSCs also produced the highest number of colony-forming unit for granulocytes and macrophages (CFU-GM) and burst-forming unit–erythroid (BFU-E) colonies in the CFU assay (Figures 5G,H). The above evidence suggested that the superior hematopoiesis-supporting potential may be attributed to higher expression of hematopoietic supporting genes in LM-MSCs.

Since MSCs show good therapeutic potential for the treatment of various diseases, it is important to evaluate the safety of MSC transplantation. Consequently, 5×10^6^ LM-MSCs were subcutaneously injected into NCG mice, and the tumorigenicity of these cells was assessed. After 2 months, we found that the control cells (undifferentiated hiPSCs) could efficiently form tumors (100%), while no evidence of tumor formation was detected in the BMSC and LM-MSC groups (Supplementary Figure S6), indicating the safety of *in vivo* transplantation of LM-MSCs.

### Global Gene Expression Profiling of Lateral Mesoderm-Mesenchymal Stem Cells

**FIGURE 6:**
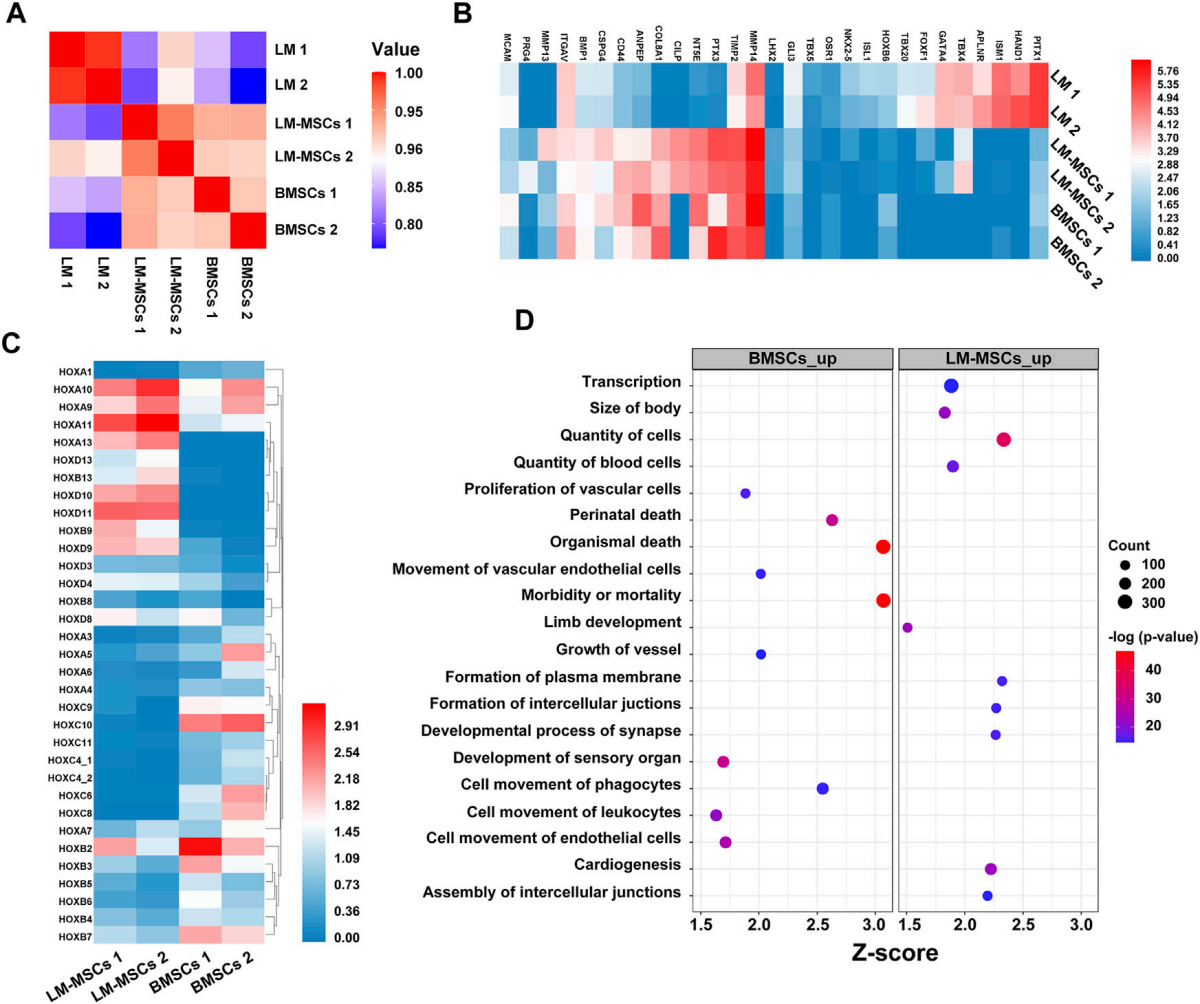
Transcriptome profiles from LM, LM-MSCs, and BMSCs. **(A)**. Pearson’s correlation coefficients of pairwise comparisons were calculated for all expressed genes in LM, LM-MSCs, and BMSCs. **(B)**. The heatmap of typical markers labeling LM, LM-MSCs and BMSCs in the RNA-Seq results was analyzed. **(C)**. The HOX gene expression pattern in LM-MSCs and BMSCs in RNA-Seq data was analyzed. **(D)**. IPA functional annotation of differentially expressed mRNAs. The dot plot of partially enriched functions. The color intensity of the nodes indicates the degree of IPA function enrichment. The horizontal axis indicates the gene ratio as the proportion of differentially expressed genes in the whole gene set. The size represents the number counts in a certain function.

To evaluate changes in the transcriptome profiles during LM-MSC differentiation, we performed RNA sequencing (RNA-Seq) in different datasets to detect genome-wide transcriptional patterns of hiPSC-derived LM (LM1, LM2), hiPSC-derived LM-MSCs (LM-MSCs 1, LM-MSCs 2), and BMSCs (BMSCs 1, BMSCs 2) (GSE182161). We first calculated the coefficients of determination (R2) for all expressed genes and revealed a high level of similarity in the gene expression profile between 2 samples at the same differentiation stage (LM 1 vs LM 2; LM-MSCs 1 vs LM-MSCs 2; R2>0.95; Figure 6A), indicating a high reproducibility of our differentiation protocol. However, the R2 value decreased when comparing the data from samples at different stages (LM vs LM-MSCs, R2 = 0.8–0.9), which suggested that significant changes in gene expression patterns occurred in cells during *in vitro* differentiation. Moreover, we determined that the LM-MSCs shared highly similar gene expression patterns with BMSCs (R2>0.90) (Figure 6A). The RNA-Seq data also showed that LM-specific transcription factors, such as HAND1 and FOXF1, were enriched in LM cells. More importantly, all the LM-MSC and BMSC samples were highly enriched in transcripts associated with MSC-specific molecules as described, including CD epitope-encoding genes such as CD29/ITGB1, CD44, CD73/NT5E, and CD166/ALCAM (Figure 6B). Increased protease (MMP/TIMP) and PTX3 expression was found in LM-MSCs and BMSCs compared to LM cells in RNA-Seq, which was further confirmed by RT-PCR assays (Supplementary Figure S7). Upregulation of these genes may be involved in the migration, invasion, ECM remodeling, and angiogenesis properties of LM-MSCs (Lu et al., 2010; Cappuzzello et al., 2016; Doni et al., 2021). In addition, RNA-Seq data indicated that LM-MSCs highly expressed HOX9-13 genes, which was consistent with the qRT-PCR results described above (Figure 6C). IPA also showed enrichment for genes involved in the quantity of blood cells, limb development, size of body and others, further suggesting that LM-MSCs possessed higher osteogenic, chondrogenic, and hematopoiesis supporting potential than BMSCs (Figure 6D). These findings were in accordance with the above experimental results.

## Discussion

In this study, we successfully established an efficient and highly reproducible differentiation protocol for the derivation of MSCs with LM origin from hPSCs. hPSC-derived LM-MSCs exhibited characteristic HOX gene profiles and shared similar properties with BMSCs in surface marker expression and immune regulation activity. Moreover, compared to BMSCs, LM-MSCs presented stronger osteogenesis and cartilage differentiation potential *in vitro* and displayed a superior ability to support bone formation and hematopoiesis *in vivo*.

The PM, IM, and LM are generated from PS cells during gastrulation (Loh et al., 2016). Thus, efficient induction of the PS from hPSCs is considered a necessary step toward acquiring mesoderm properties *in vitro*. Preliminary studies have shown that TGF-β superfamily members (including Activin/Nodal and BMP), fibroblast growth factors (FGFs), and WNT signaling are key regulators determining PS cell fate during embryogenesis (Wang and Chen, 2016). Most studies have used high doses of Activin A alone or in synergism with BMP, FGF or WNT activators to induce PS differentiation from hPSCs. However, only a few studies have shown that the activation of the WNT signaling pathway alone was sufficient to direct hPSCs to differentiate into the PS *in vitro* (Wang and Chen, 2016). Sasha et al. reported that significant induction of TBXT transcripts and protein expression could be achieved in hPSCs when treated with only one molecule, CHIR, for 36 h. These researchers also found that 8 μM CHIR yielded higher TBXT expression than 3 μM CHIR, as shown by the immunostaining assay. However, this group did not quantify the PS differentiation efficiency (the proportion of TBXT + cells) induced by CHIR alone. Bone et al. showed that when cultured in mTeSR medium supplemented with 2 μM CHIR, hPSCs started to differentiate into PS cells, and TBXT protein expression was detected on Day 3 and remained elevated until Day 7. Nonetheless, TGF-β and FGF signaling was also activated in their study since the basal medium, mTeSR, contained TGF-β1 and FGF2 (Arnold and Robertson, 2009; Bone et al., 2011). Lam et al. also demonstrated that treatment with 5 μM CHIR for 24 h resulted in 98.7 ± 1.3% of the cells expressing TBXT during hPSC differentiation (Lam et al., 2014). Herein, we analyzed the time-dependent and dosage-dependent effects of CHIR on hPSC differentiation under serum-free conditions. We found that the mRNA levels of TBXT and MIXL1 increased markedly when cells were treated with 3 μM CHIR for 24 h; however, the pluripotency marker NANOG was still expressed in some of the differentiated hPSCs, as detected by qRT-PCR and immunocytochemistry. Our results also showed that hPSCs could successfully exit pluripotency and differentiate toward TBXT+/MIXL1+ PS cells with high efficiency when treated with 3 μM CHIR for 2 days. Moreover, the expression of TBXT began to decline when hPSCs were treated with CHIR for more than 2 days. Accordingly, we demonstrated that activation of WNT signaling with 3 μM CHIR for 2 days could efficiently induce hPSCs to differentiate into the PS.

Previous studies have shown that WNT signaling is vital for mesoderm specification and separation *in vivo*, and blocking WNT signals in ES cells abrogated the differentiation of FLK1-positive mesoderm *in vitro* (Lindsley et al., 2006; Tanaka et al., 2011). Several protocols have been described for obtaining cells with the characteristics of LM. Kyle et al. found that BMP and WNT signals help drive the bifurcation of lateral versus paraxial mesoderm subtypes from the PS. The authors showed that BMP induces, whereas WNT inhibits, LM from the PS. A 2-days treatment with A8301, BMP4, and C59 (a WNT inhibitor) could direct PS differentiation into HAND1+/FOXF1+ LM cells with an efficiency of 98.1–100% (Loh et al., 2016). Liu et al. established an efficient protocol for the differentiation of LM from hPSCs by the combination of FGF2, BMP4, the ROCK inhibitor Y27632, and follistatin (Liu et al., 2020).

In this study, we first showed that prolonged exposure to CHIR alone (4–8 days) resulted in a heterogeneous population of differentiated cells containing less than 50% LM progenitors. These results suggested that CHIR was not sufficient to induce homogeneous LM cells in the absence of additional exogenous factors. James et al. reported that high levels of BMP repressed both paraxial and intermediate mesoderm gene expression but activated lateral plate genes during chick embryo development (James and Schultheiss, 2005). Indeed, our results confirmed that the addition of BMP to CHIR-containing induction medium could promote the LM fate (nearly 100%) and inhibit the development of intermediate or paraxial mesoderm from the PS *in vitro*. Interestingly, our results indicated that BMP7 had a stronger promoting effect on the expression of LM-related genes than BMP4. The underlying molecular mechanisms need to be further elucidated. Taken together, our results demonstrated that LM progenitors could be readily generated from the PS following treatment with BMP7 and CHIR.

The heterogeneous properties of MSCs have been considered the cause of their inconsistent therapeutic efficacy in many clinical trials. MSCs have multiple developmental origins, including the neural crest (neural ectoderm), mesoderm, and trophoblast (Jiang et al., 2019). Therefore, minimization of the heterogeneity of MSCs by better defining developmental origins may help to stabilize the treatment effects in the clinical translation of MSCs. Previous studies have suggested that an MSC-like population exists during both LM differentiation and homeostatic maintenance of LM-derived tissues (Sheng, 2015). However, to our knowledge, few studies have reported protocols for the derivation of MSCs from LM. Two studies have successfully derived MSCs through the intermediate stages of LM and via subsequent mesenchymoangioblasts of hPSCs (Vodyanik et al., 2010; Ozay et al., 2019). However, these MSCs may represent a mixed population including limb MSCs and pericytes, since the mesenchymoangioblast is the earliest precursor of endothelial cells, mesenchymal cells, and pericytes originating from KDR + hematopoietic mesoderm. Liu et al. established an efficient protocol for the differentiation of skeletal mesenchymal stromal cells through LM from hPSCs. Their results revealed that hPSC-MSCs showed robust mesenchymal trilineage differentiation potential, similar to primary BMSCs. Nevertheless, they did not detect the expression of members of the HOX gene family, immune modulatory function, or hematopoiesis-supporting activity of these skeletal MSCs (Liu et al., 2020). In our study, we successfully acquired LM-MSCs in chemically defined and animal component-free MSC medium, which meets the 2006 ISCT guidelines for MSC characterization (Dominici et al., 2006). We showed that LM-MSCs possessed a stronger osteogenic differentiation capacity than BMSCs *in vitro* and *in vivo*. We hypothesized that the *in vivo* chondrogenic differentiation ability of LM-MSCs may also be stronger than that of BMSCs, since the *in vitro* differentiation assay showed a similar trend and the RNA-Seq data showed that genes of “limb development” were highly enriched in LM-MSCs compared with BMSCs, which needs further experimental verification. LM-MSCs also displayed HOX expression patterns similar to those of skeletal progenitor cells in the vertebrate limb (Cohn et al., 1997). We further confirmed that LM-MSCs resembled primary BM-BMSCs with immunomodulatory properties, as LM-MSCs could efficiently inhibit the proliferation of CD3^+^ T cells and suppress the percentages of CD3^+^ T cells and macrophages that produced TNF-α and/or IFN-γ. Intriguingly, we showed that LM-MSCs have a superior ability to support hematopoiesis. Moreover, analysis of RNA-Seq data demonstrated that the R2 between different batches of LM-MSCs from the same hPSC line (LM-MSCs 1 vs LM-MSCs 2) was higher than 0.95, indicating that a relatively homogeneous cell population could be obtained by our differentiation protocol. These results suggested that functional LM-MSCs were successfully generated by a chemically defined MSC medium.

In conclusion, we developed a simple, efficient, and chemically defined protocol for the derivation of MSCs with LM origin from hPSCs. Our findings also supported the critical role of the BMP and WNT signaling pathways in LM commitment from PS cells. These LM-MSCs derived from hPSCs may be valuable in elucidating the biological characteristics of their *in vivo* counterparts. LM-MSCs may also represent a new source in cell replacement therapy for bone/cartilage defects and hematopoietic diseases.

## Data Availability

The datasets presented in this study can be found in online repositories. The names of the repository/repositories and accession number(s) can be found below: https://www.ncbi.nlm.nih.gov/geo/ GSE182161.

## Abbreviations

MSCs: mesenchymal stem cells; hPSCs: human pluripotent stem cells; hESCs: human embryonic stem cells; hiPSCs: human induced pluripotent stem cells; BMSCs: bone marrow mesenchymal stem cells; HOX: homeodomain transcription factor; LM: lateral mesoderm; NEAA: nonessential amino acids; ITS: insulin-transferrin-selenium; L-GLU: l-glutamic acid; BMP-7: bone morphogenetic protein-7; qRT-PCR: quantitative reverse transcription-polymerase chain reaction; LM-MSCs: LM-derived MSCs; FCM: flow cytometry; CCK8: Cell Counting Kit-8; HA/TCP: hydroxyl-apatite/tricalcium phosphate ceramic powder; EDTA: ethylenediaminetetraacetic acid; H&E: hematoxylin and eosin; PBMCs: peripheral blood mononuclear cells; CFSE: carboxyfluorescein succinimidyl ester; DAPI: 4′,6-diamino-2-phenylindole; RNA-Seq: RNA sequencing; PCA: principal component analysis; TBXT: brachyury; CHIR: CHIR99021; TGF-β1: transforming growth factor-β1; PM: paraxial mesoderm; IM: intermediate mesoderm; TIMP: tissue inhibitors of metalloproteinases; TNF-α: tumor necrosis factor α; IFN-γ: interferon γ.
