# Supplementary figures and images for: Lateral Mesoderm-Derived Mesenchymal Stem Cells With Robust Osteochondrogenic Potential and Hematopoiesis-Supporting Ability
Source: Front Mol Biosci. 2022 Apr 28;9:767536. doi: 10.3389/fmolb.2022.767536 (PMC9095820; doi:10.3389/fmolb.2022.767536)

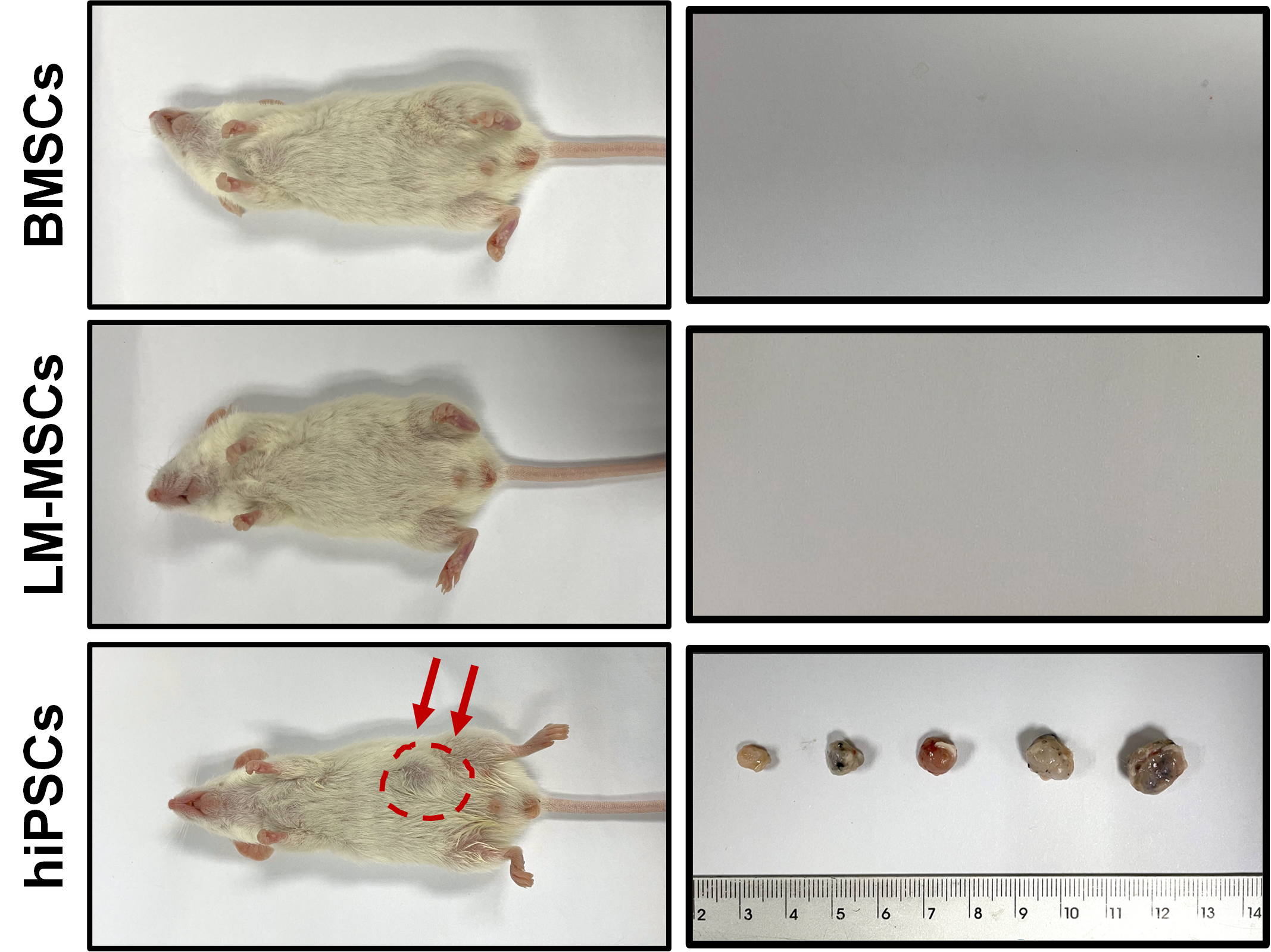

Supplement: Supplementary file 1 [file Image6.TIF]

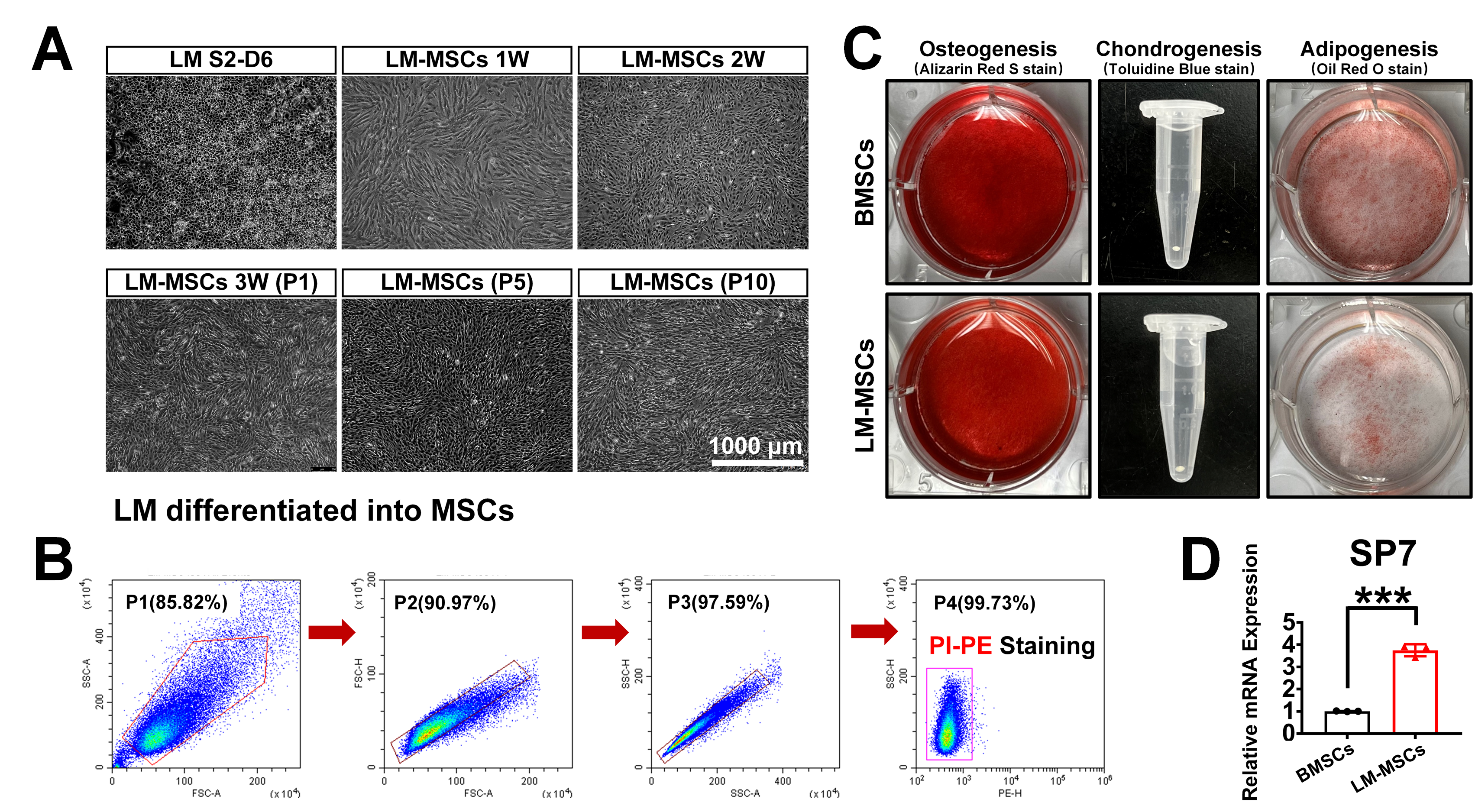

Supplement: Supplementary file 3 [file Image3.tif]

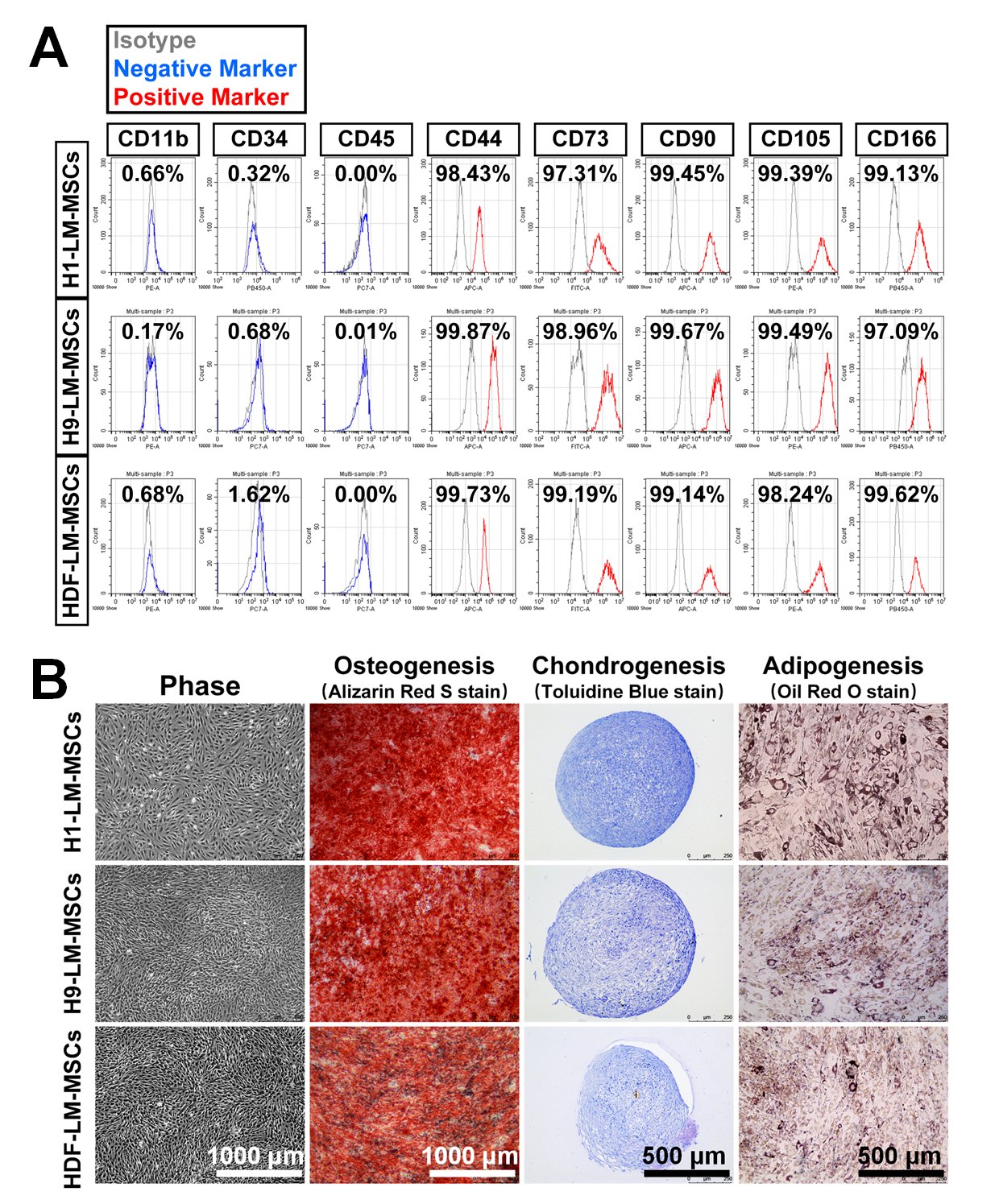

Supplement: Supplementary file 4 [file Image4.TIF]

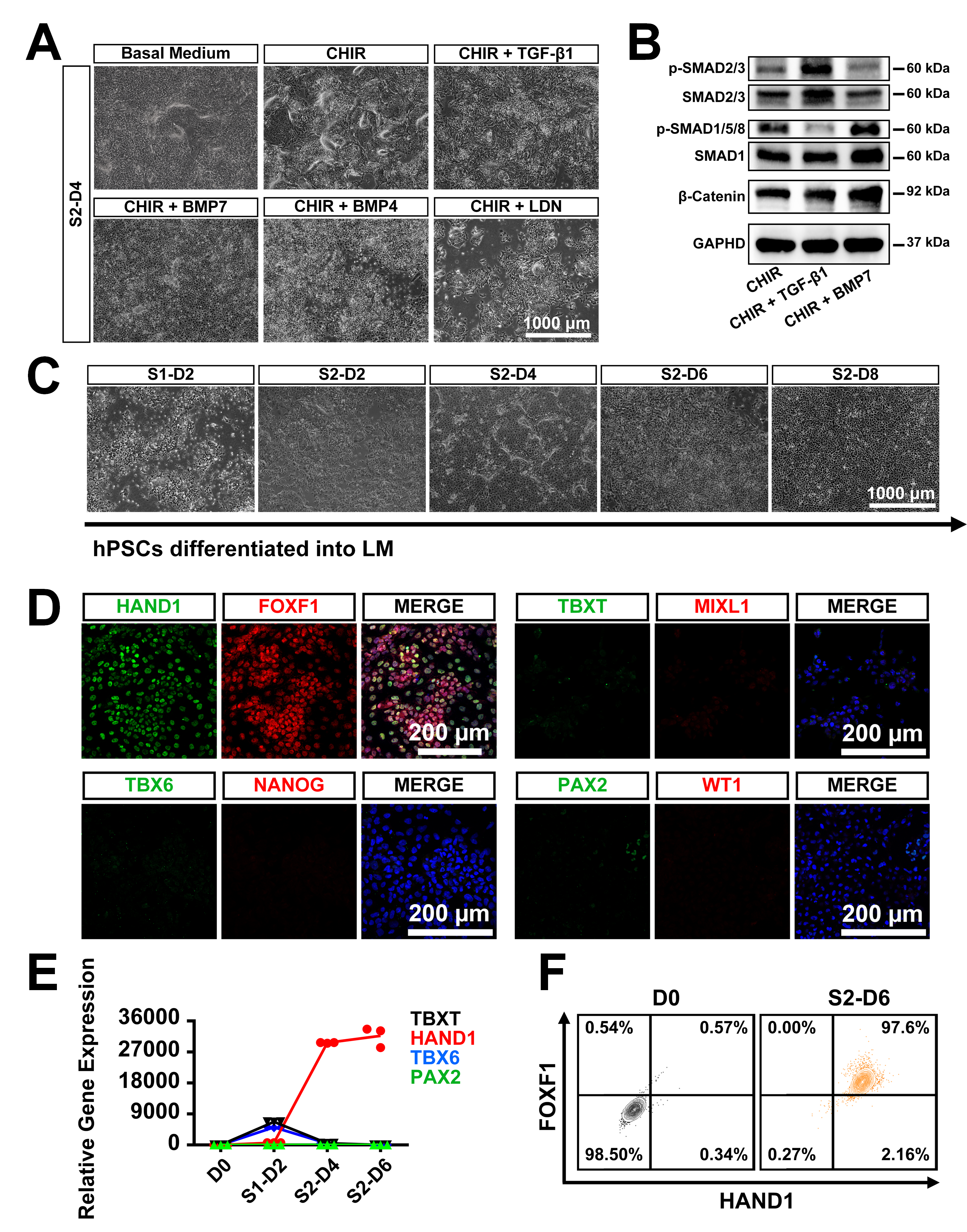

Supplement: Supplementary file 5 [file Image2.TIF]

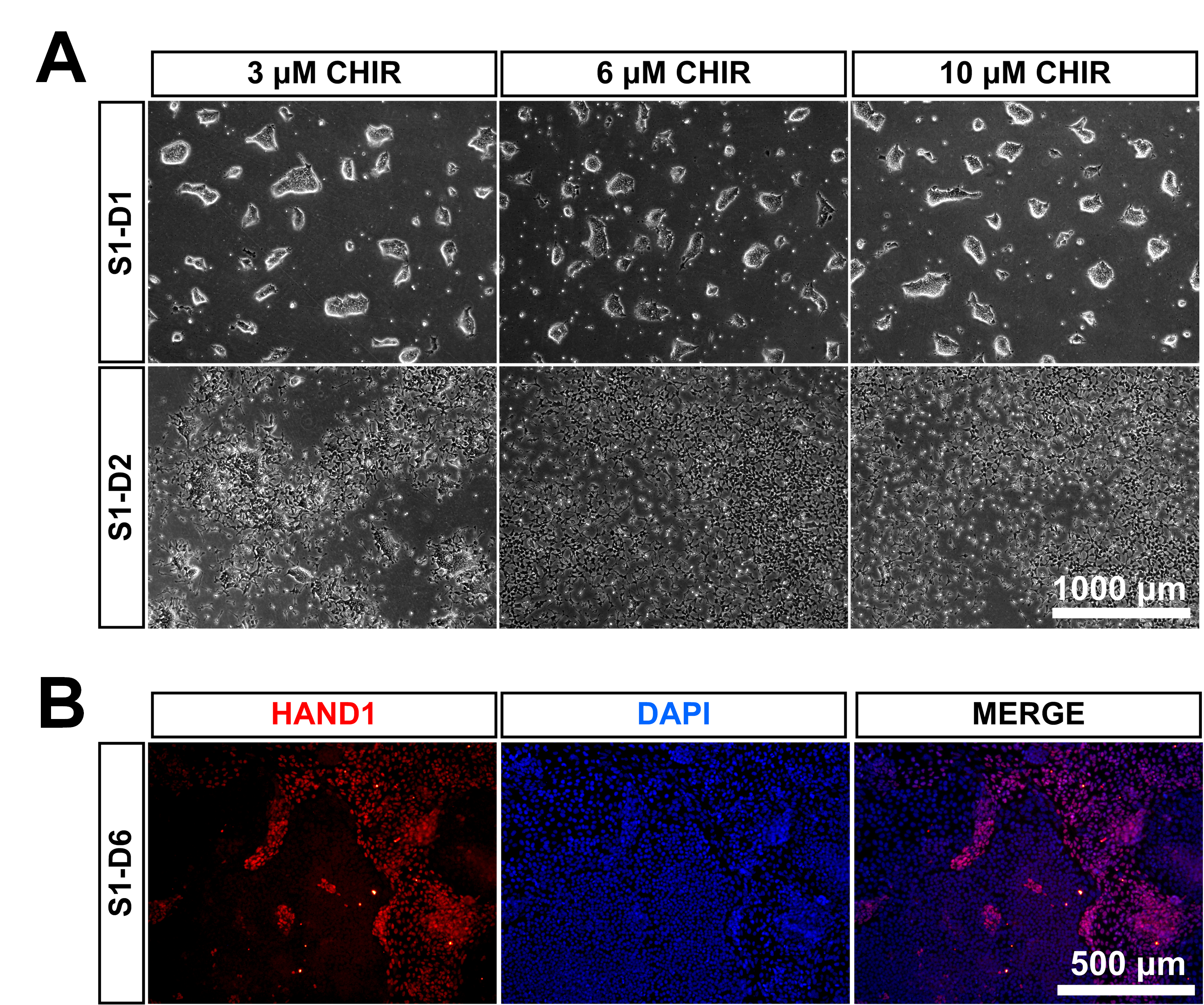

Supplement: Supplementary file 6 [file Image1.TIF]

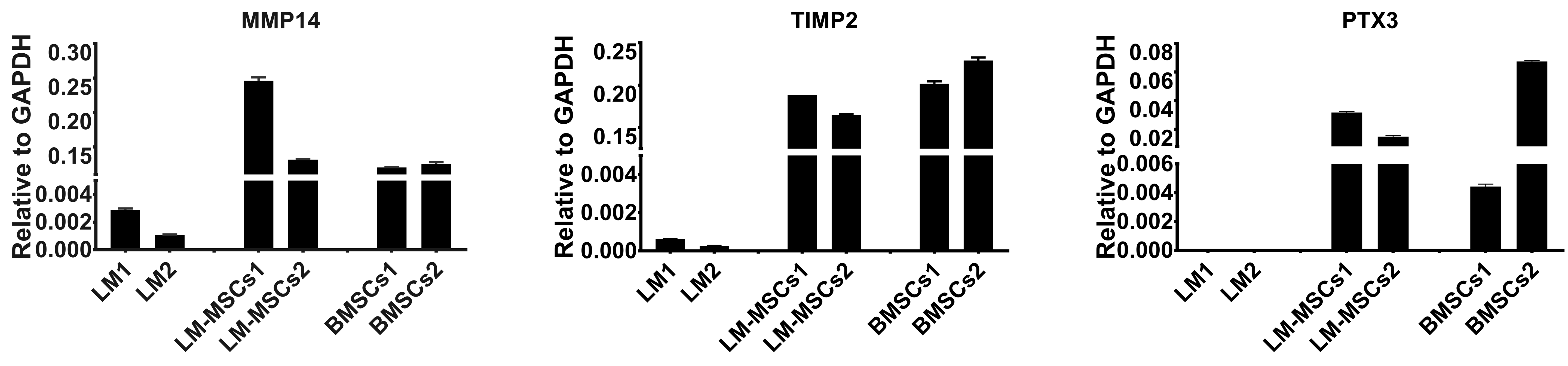

Supplement: Supplementary file 7 [file Image7.TIF]

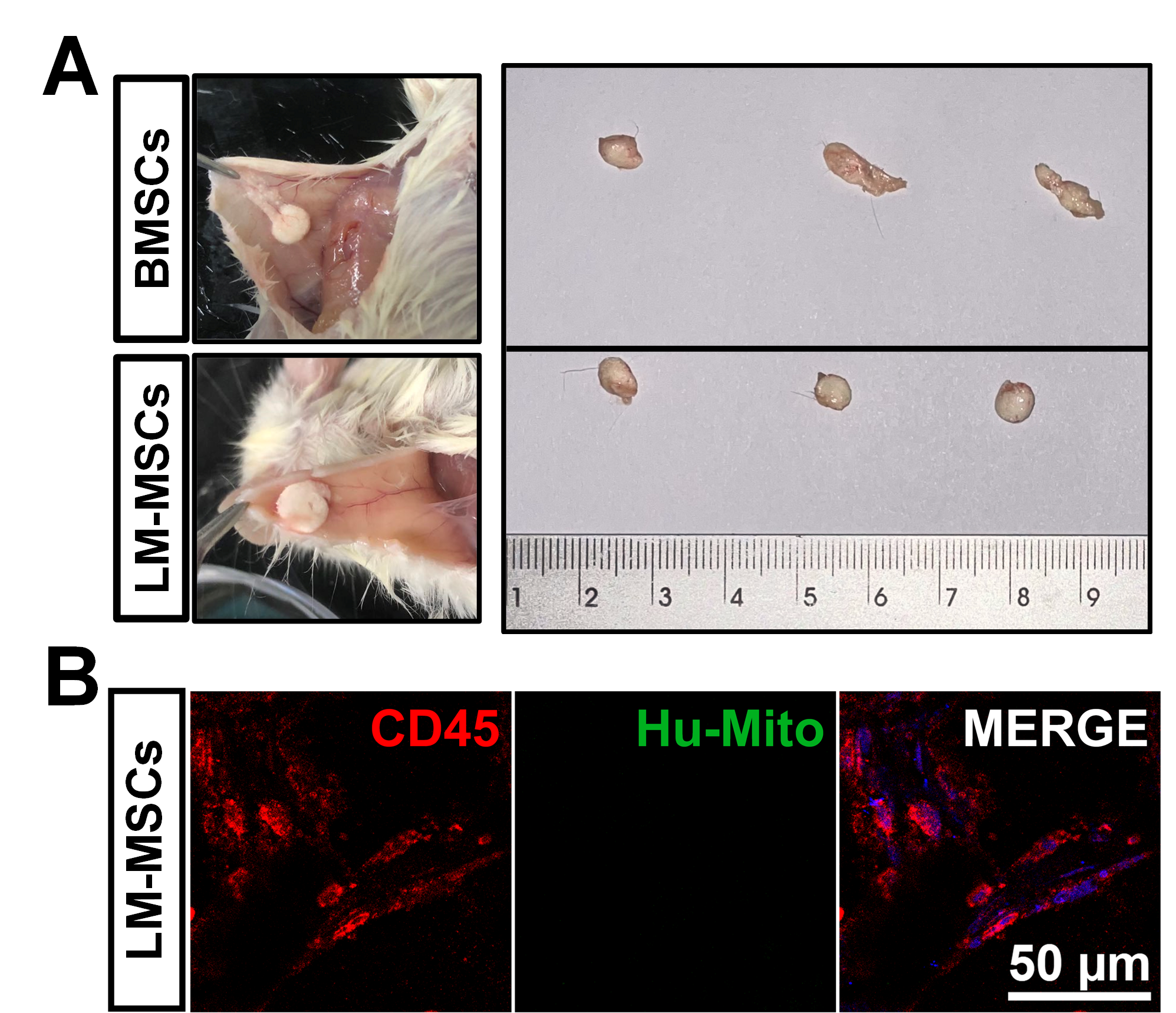

Supplement: Supplementary file 11 [file Image5.TIF]
