# Supplementary material for: Lateral Mesoderm-Derived Mesenchymal Stem Cells With Robust Osteochondrogenic Potential and Hematopoiesis-Supporting Ability
Source: Front Mol Biosci. 2022 Apr 28;9:767536. doi: 10.3389/fmolb.2022.767536 (PMC9095820; doi:10.3389/fmolb.2022.767536)
Supplement: Supplementary file 2 [file Table1.DOCX]

# Supplementary figure legends

**Supplementary Figure 1. Primitive streak differentiation from hPSCs.**

1. Phase-contrast images of hiPSCs when treated with different concentrations (3, 6, and 10 μM) of CHIR99021 at stage 1 (S1) for 1 days (S1-D1) and 2 days (S1-D2). Scale bar: 1000 μm.
2. Immunostaining for expression of the lateral mesoderm marker HAND1 when hiPSCs were treated with 3 μM CHIR99021 for 6 days (S1-D6). Scale bar: 500 μm.

**Supplementary Figure 2. Differentiation of lateral mesoderm from primitive streak.**

1. Phase-contrast images of primitive streak cells that were treated with different factors (basal medium, CHIR, CHIR+TGFβ1, CHIR+BMP7, CHIR+BMP4, or CHIR+LDN192189) at stage 2 for 4 days (S2-D4). Scale bar: 1000 μm.
2. Western blotting analysis for the signaling pathway molecules (β-Catenin/SMAD) involved in the determination of LM fate from the PS *in vitro*.
3. Phase-contrast images during lateral mesoderm differentiation from hiPSCs when treated with 3 μM CHIR99021 at stage 1 (S1) for 2 days (S1-D2) followed by 3 μM CHIR99021 and 100 ng/mL BMP7 at stage 2 (S2) for 8 days (S2-D8). Scale bar: 1000 μm.
4. Immunostaining images for the markers of pluripotency (NANOG), primitive streak (TBXT), lateral mesoderm (HAND1; FOXF1), paraxial mesoderm (TBX6, MIXL1), and intermediate mesoderm (PAX2, WT1) in hESC- (H1-) derived lateral mesoderm at stage 2 on day 6 (S2-D6). Scale bar: 200 μm.
5. qRT-PCR analysis for the markers of primitive streak (TBXT), lateral mesoderm (HAND1), paraxial mesoderm (TBX6), and intermediate mesoderm (PAX2) during LM differentiation from H1 cell line.
6. Quantification of H1-derived lateral mesoderm (HAND1; FOXF1) cells on S2-D6 by FCM.

**Supplementary Figure 3. Derivation and characterization of LM-MSCs derived from hiPSCs.**

1. Phase-contrast images of the MSC differentiation process when hiPSC-derived LM cells were cultured in serum-free MSC medium. Scale bar: 1000 μm.
2. The sample gating strategy during FCM was provided. Cell debris (SSC-A vs FSC-A), clumps or doublets (FSC-H vs FSC-A; SSC-H vs SSC-A), and dead cells (PI positive cells) were excluded.
3. The general staining images of oil red O staining and alizarin red s staining, and photographs of cartilage micromass after *in vitro* multilineage differentiation of LM-MSCs and BMSCs.
4. qRT-PCR analysis for the gene expression of osteogenic (SP7) of LM-MSCs and BMSCs.

**Supplementary Figure 4. Characterization of LM-MSCs derived from hESCs (H1, H9), and HDF-hiPSCs.**

1. Flow cytometry for detection of typical MSC surface markers in LM-MSCs derived from hESCs (H1, H9) and HDF-hiPSCs.
2. Phase-contrast images of LM-MSCs derived from hESCs (H1, H9) and HDF-hiPSCs. The osteogenic, chondrogenic, and adipogenic differentiation potentials of these LM-MSCs were verified by Alizarin Red S staining (Scale bar: 1000 μm), toluidine blue staining (Scale bar: 500 μm), and oil red O staining (Scale bar: 500 μm), respectively.

**Supplementary Figure 5. *In vivo* bone formation of LM-MSCs derived from hiPSCs.**

1. Photos of *in vivo* bone formation samples of hiPSC-derived LM-MSCs and BMSCs.
2. Co-immunofluorescence staining of hematopoietic cell clusters in LM-MSC implants using anti-mouse CD45 antibody and anti-human mitochondria antibody.

**Supplementary Figure 6. *In vivo* tumor formation assay of LM-MSCs derived from hiPSCs.**

Tumor formation assay of hiPSC-derived LM-MSCs, BMSCs, and hiPSCs (n=5). we found that undifferentiated hiPSCs could efficiently form tumors (100%), while no evidence of tumor formation was detected in the BMSC group and LM-MSC group.

**Supplementary Figure 7. qRT-PCR detection of the gene expression of MMP14, TIMP2 and PTX3 in LM-MSCs and BMSCs**.
