# Supplementary material for: Lateral Mesoderm-Derived Mesenchymal Stem Cells With Robust Osteochondrogenic Potential and Hematopoiesis-Supporting Ability
Source: Front Mol Biosci. 2022 Apr 28;9:767536. doi: 10.3389/fmolb.2022.767536 (PMC9095820; doi:10.3389/fmolb.2022.767536)
Supplement: Supplementary file 8 [file Table2.DOCX]

**Supplementary Table 2. Antibodies used in FCM**

| Antigen | Label | Company | Cat. No. |
| --- | --- | --- | --- |
| Anti-human HLA-DR | V500 | BD Pharmingen | 561224 |
| Anti-human CD11b | PE | BD Pharmingen | 555388 |
| Anti-human CD19 | FITC | Invitrogen | 11-0199-42 |
| Anti-human CD34 | PECY7 | BD Pharmingen | 560710 |
| Anti-human CD34 | FITC | Invitrogen | 11-0349-42 |
| Anti-human CD45 | PECY7 | BD Pharmingen | 557748 |
| Anti-human CD44 | APC | BD Pharmingen | 559942 |
| Anti-human CD73 | FITC | BD Pharmingen | 561254 |
| Anti-human CD90 | APC | BD Pharmingen | 559869 |
| Anti-human CD105 | PE | Invitrogen | 12-1057-42 |
| Anti-human CD166 | BV421 | BD Pharmingen | 562936 |
| Anti-human IFN-γ | PECY7 | BD Pharmingen | 557844 |
| Anti-human TNF-α | PE | BD Pharmingen | 554513 |
