# Supplementary material for: Lateral Mesoderm-Derived Mesenchymal Stem Cells With Robust Osteochondrogenic Potential and Hematopoiesis-Supporting Ability
Source: Front Mol Biosci. 2022 Apr 28;9:767536. doi: 10.3389/fmolb.2022.767536 (PMC9095820; doi:10.3389/fmolb.2022.767536)
Supplement: Supplementary file 9 [file Table3.DOCX]

**Supplementary Table 3. Primers used for qRT-PCR**

| **Gene** | **Direction** | **Sequences** |
| --- | --- | --- |
| *GAPDH* | Forward | CACCGTCAAGGCTGAGAACG |
|  | Reverse | GCCCCACTTGATTTTGGAGG |
| *NANOG* | Forward | TTTGTGGGCCTGAAGAAAACT |
|  | Reverse | AGGGCTGTCCTGAATAAGCAG |
| *OCT4* | Forward | CAGTGCCCGAAACCCACAC |
|  | Reverse | GGAGACCCAGCAGCCTCAAA |
| *SOX1* | Forward | CAGTACAGCCCCATCTCCAAC |
|  | Reverse | GCGGGCAAGTACATGCTGA |
| *PAX6* | Forward | TGGGCAGGTATTACGAGACTG |
|  | Reverse | ACTCCCGCTTATACTGGGCTA |
| *SOX17* | Forward | GTGGACCGCACGGAATTTG |
|  | Reverse | GGAGATTCACACCGGAGTCA |
| *FOXA2* | Forward | GGAGCAGCTACTATGCAGAGC |
|  | Reverse | CGTGTTCATGCCGTTCATCC |
| *TBXT* | Forward | TGCTTCCCTGAGACCCAGTT |
|  | Reverse | GATCACTTCTTTCCTTTGCATCAAG |
| *MIXL1* | Forward | GGTACCCCGACATCCACTTG |
|  | Reverse | TAATCTCCGGCCTAGCCAAA |
| *TBX6* | Forward | AAGTACCAACCCCGCATACA |
|  | Reverse | TAGGCTGTCACGGAGATGAA |
| *MSGN1* | Forward | CGGAATTACCTGCCACCTGT |
|  | Reverse | GGTCTGTGAGTTCCCCGATG |
| *HAND1* | Forward | GTGCGTCCTTTAATCCTCTTC |
|  | Reverse | GTGAGAGCAAGCGGAAAAG |
| *FOXF1* | Forward | AGCAGCCGTATCTGCACCAGAA |
|  | Reverse | CTCCTTTCGGTCACACATGCTG |
| *PAX2* | Forward | AGATTCCCAGAGTGGTGTGG |
|  | Reverse | GGGTATGTCTGTGTGCCTGA |
| *PAX8* | Forward | GCAACCATTCAACCTCCCTA |
|  | Reverse | CTGCTGCTGCTCTGTGAGTC |
| *LPL* | Forward | ACAAGAGAGAACCAGACTCCAA |
|  | Reverse | AGGGTAGTTAAACTCCTCCTCC |
| *PPARγ* | Forward | ACCAAAGTGCAATCAAAGTGGA |
|  | Reverse | ATGAGGGAGTTGGAAGGCTCT |
| *ADIPOQ* | Forward | CAAGTGCGTCCAGAGAAGATT |
|  | Reverse | TCTGACCGATGGCAGGAAAAA |
| *AP2* | Forward | ACTGGGCCAGGAATTTGACG |
|  | Reverse | CTCGTGGAAGTGACGCCTT |
| *ALP* | Forward | AGCACTCCCACTTCATCTGGAA |
|  | Reverse | GAGACCCAATAGGTAGTCCACATTG |
| *COL1A1* | Forward | CAGCCGCTTCACCTACAGC |
|  | Reverse | TTTTGTATTCAATCACTGTCTTGCC |
| *OCN* | Forward | CAGCGAGGTAGTGAAGAGA |
|  | Reverse | GAAAGCCGATGTGGTCAG |
| *OPN* | Forward | AGATGGGTCAGGGTTTAGCC |
|  | Reverse | CATCACCTGTGCCATACCAG |
| *SP7* | Forward | CCTCTGCGGGACTCAACAAC |
|  | Reverse | AGCCCATTAGTGCTTGTAAAGG |
| *ACAN* | Forward | CCCCTGCTATTTCATCGACCC |
|  | Reverse | GACACACGGCTCCACTTGAT |
| *COL2A1* | Forward | GGCAATAGCAGGTTCACGTACA |
|  | Reverse | CGATAACAGTCTTGCCCCACTT |
| *RUNX2* | Forward | CCGCCTCAGTGATTTAGGGC |
|  | Reverse | GGGTCTGTAATCTGACTCTGTCC |
| *SOX9* | Forward | CGTCAACGGCTCCAGCAAGAACAA |
|  | Reverse | GCCGCTTCTCGCTCTCGTTCAGAAGT |
| *IL6* | Forward | ACTCACCTCTTCAGAACGAATTG |
|  | Reverse | CCATCTTTGGAAGGTTCAGGTTG |
| *IL8* | Forward | ACTGAGAGTGATTGAGAGTGGAC |
|  | Reverse | AACCCTCTGCACCCAGTTTTC |
| *IDO* | Forward | TCTCATTTCGTGATGGAGACTGC |
|  | Reverse | GTGTCCCGTTCTTGCATTTGC |
| *CCL2* | Forward | CAGCCAGATGCAATCAATGCC |
|  | Reverse | TGGAATCCTGAACCCACTTCT |
| *PDL1* | Forward | TGCCGACTACAAGCGAATTACTG |
|  | Reverse | CTGCTTGTCCAGATGACTTCGG |
| *TSG6* | Forward | TCACCTACGCAGAAGCTAAGGC |
|  | Reverse | TCCAACTCTGCCCTTAGCCATC |
| *HOXA9* | Forward | GTCCAAGGCGACGGTGTTT |
|  | Reverse | CCGACAGCGGTTCAGGTTTA |
| *HOXB9* | Forward | GAGAGGCCGGATCAAACCAA |
|  | Reverse | CTACGGTCCCTGGTGAGGTA |
| *HOXC9* | Forward | AGCACAAAGAGGAGAAGGC |
|  | Reverse | CGTCTGGTACTTGGTGTAGG |
| *HOXD9* | Forward | GGACTCGCTTATAGGCCATGA |
|  | Reverse | GCAAAACTACACGAGGCGAA |
| *HOXA10* | Forward | CTCGCCCATAGACCTGTGG |
|  | Reverse | GTTCTGCGCGAAAGAGCAC |
| *HOXD10* | Forward | GGACTCGCTTATAGGCCATGA |
|  | Reverse | GCAAAACTACACGAGGCGAA |
| *HOXA11* | Forward | CCCGCAGTCTCGTCCAATTT |
|  | Reverse | AGGCTGTCTCGAAAAACTGGT |
| *HOXD11* | Forward | TCGACCAGTTCTACGAGGCA |
|  | Reverse | AAAAACTCGCGTTCCAGTTCG |
| *HOXA13* | Forward | CTGGCATTTTCCTCTCCCGAA |
|  | Reverse | ATTACCATCTAACGCAGTGTCC |
| *HOXD13* | Forward | CTTCGGCAACGGCTACTACAG |
|  | Reverse | TGACACGTCCATGTACTTCTCC |
| *CXCL12* | Forward | TGGGCTCCTACTGTAAGGGTT |
|  | Reverse | TTGACCCGAAGCTAAAGTGG |
| *VCAM1* | Forward | GTCTCCAATCTGAGCAGCAA |
|  | Reverse | TGAGGATGGAAGATTCTGGA |
| *MCP1* | Forward | AGAATCACCAGCAGCAAGTGTCC |
|  | Reverse | TCCTGAACCCACTTCTGCTTGG |
| *KITLG* | Forward | AATCCTCTCGTCAAAACTGAAGG |
|  | Reverse | CCATCTCGCTTATCCAACAATGA |
| *FLT3L* | Forward | CTATGCATCCTCTGGCTGGT |
|  | Reverse | CTGCTGCTTGTGGAGCACT |
| *ANGPT1* | Forward | GCCATCTCCGACTTCATGTT |
|  | Reverse | CTGCAGAGAGATGCTCCACA |
| *MMP14* | Forward | CATCTGTGACGGGAACTTTGA |
|  | Reverse | GGCAGTGTTGATGGACGCA |
| *TIMP2* | Forward | GCTGCGAGTGCAAGATCAC |
|  | Reverse | TGGTGCCCGTTGATGTTCTTC |
| *PTX3* | Forward | CATCTCCTTGCGATTCTGTTTTG |
|  | Reverse | CCATTCCGAGTGCTCCTGA |
