# Supplementary material for: Lateral Mesoderm-Derived Mesenchymal Stem Cells With Robust Osteochondrogenic Potential and Hematopoiesis-Supporting Ability
Source: Front Mol Biosci. 2022 Apr 28;9:767536. doi: 10.3389/fmolb.2022.767536 (PMC9095820; doi:10.3389/fmolb.2022.767536)
Supplement: Supplementary file 10 [file Table4.DOCX]

**Supplementary Table 3. Antibodies used in Immunofluorescence Staining and Western blot**

| **Antigen** | **Host** | **Dilution** | **Company** | **Cat. No.** |
| --- | --- | --- | --- | --- |
| NANOG | Rabbit | 1:400 | Cell signaling technology | 4903S |
| SOX17 | Goat | 1:100 | R&D | AF1924 |
| PAX6 | rabbit | 1:200 | Abcam | ab195045 |
| TBXT | Goat | 1:400 | R&D | AF2085 |
| MIXL1 | Rabbit | 1:400 | Proteintech Group | 22772-1-AP |
| TBX6 | Goat | 1:400 | R&D | AF4744 |
| HAND1 | Goat | 1:400 | R&D | AF3168 |
| FOXF1 | Rabbit | 1:200 | Abcam | ab168383 |
| PAX2 | Goat | 1:400 | R&D | AF3364 |
| WT1 | Rabbit | 1:400 | Cell Signaling Technology | 83535 |
| Anti-mouse CD45 | Rat | 1:100 | eBioscience | 12-0451-82 |
| Anti-human Mitochondria | Mouse | 1:100 | Merck Millipore | MAB1273 |
| PPAR γ | Rabbit | 1:1000 | Cell signaling technology | 2435T |
| COL1A1 | Mouse | 1:1000 | Invitrogen | PA5-29569 |
| COL2A1 | Rabbit | 1:1000 | Abcam | ab34712 |
| OCN | Mouse | 1:400 | R&D | MAB1419 |
| OPG | Rabbit | 1:400 | Merck Millipore | ABC463 |
| VCAM-1 | Rabbit | 1:1000 | Cell signaling technology | 13662S |
| GAPDH | Rabbit | 1:1000 | Cell signaling technology | 2118S |
| α-Tubulin | Mouse | 1:1000 | Cell signaling technology | 3873 |
| β-Catenin | Rabbit | 1:1000 | Cell signaling technology | 8480S |
| SMAD1 | Rabbit | 1:1000 | Cell signaling technology | 6944T |
| p-SMAD1/5/8 | Rabbit | 1:1000 | Cell signaling technology | 9511S |
| SMAD2/3 | Rabbit | 1:1000 | Cell signaling technology | 8828S |
| p-SMAD2/3 | Rabbit | 1:1000 | Cell signaling technology | 8685S |
